# Is Nucleoredoxin a Master Regulator of Cellular Redox Homeostasis? Its Implication in Different Pathologies

**DOI:** 10.3390/antiox11040670

**Published:** 2022-03-30

**Authors:** Osiris Germán Idelfonso-García, Brisa Rodope Alarcón-Sánchez, Verónica Rocío Vásquez-Garzón, Rafael Baltiérrez-Hoyos, Saúl Villa-Treviño, Pablo Muriel, Héctor Serrano, Julio Isael Pérez-Carreón, Jaime Arellanes-Robledo

**Affiliations:** 1 Laboratory of Liver Diseases, National Institute of Genomic Medicine–INMEGEN, Mexico City 14610, Mexico; oidelfonso@inmegen.edu.mx (O.G.I.-G.); brisa.alarcon@cinvestav.mx (B.R.A.-S.); jiperez@inmegen.gob.mx (J.I.P.-C.); 2 Department of Health Sciences, Metropolitan Autonomous University-Iztapalapa Campus, Mexico City 09340, Mexico; hser@xanum.uam.mx; 3 Department of Cell Biology, Center for Research and Advanced Studies of the National Polytechnic Institute–CINVESTAV-IPN, Mexico City 07360, Mexico; svilla@cell.cinvestav.mx; 4 Laboratory of Fibrosis and Cancer, Faculty of Medicine and Surgery, ‘Benito Juárez’ Autonomous University of Oaxaca–UABJO, Oaxaca 68020, Mexico; vrvasquezga@conacyt.mx (V.R.V.-G.); rbaltierrezho@conacyt.mx (R.B.-H.); 5 Directorate of Cátedras, National Council of Science and Technology–CONACYT, Mexico City 03940, Mexico; 6 Laboratory of Experimental Hepatology, Department of Pharmacology, Center for Research and Advanced Studies of the National Polytechnic Institute–CINVESTAV-IPN, Mexico City 07360, Mexico; pmuriel@cinvestav.mx

**Keywords:** oxidative stress, oxidoreductase, redox regulation, thioredoxin

## Abstract

Nucleoredoxin (NXN), an oxidoreductase enzyme, contributes to cellular redox homeostasis by regulating different signaling pathways in a redox-dependent manner. By interacting with seven proteins so far, namely disheveled (DVL), protein phosphatase 2A (PP2A), phosphofructokinase-1 (PFK1), translocation protein SEC63 homolog (SEC63), myeloid differentiation primary response gene-88 (MYD88), flightless-I (FLII), and calcium/calmodulin-dependent protein kinase II type alpha (CAMK2A), NXN is involved in the regulation of several key cellular processes, including proliferation, organogenesis, cell cycle progression, glycolysis, innate immunity and inflammation, motility, contraction, protein transport into the endoplasmic reticulum, neuronal plasticity, among others; as a result, NXN has been implicated in different pathologies, such as cancer, alcoholic and polycystic liver disease, liver fibrogenesis, obesity, Robinow syndrome, diabetes mellitus, Alzheimer’s disease, and retinitis pigmentosa. Together, this evidence places NXN as a strong candidate to be a master redox regulator of cell physiology and as the hub of different redox-sensitive signaling pathways and associated pathologies. This review summarizes and discusses the current insights on NXN-dependent redox regulation and its implication in different pathologies.

## 1. Introduction

Thioredoxins (TRX) are small thiol-oxidoreductase enzymes that regulate cellular redox homeostasis, and their functioning depend on the cyclic reduction-oxidation of a single disulfide group in the enzymes [1]. TRX superfamily includes conventional TRX, glutaredoxin, protein disulfide isomerase and NXN [2]. *NXN* gene was first cloned and overexpressed in cell cultures and, since NXN was identified as a nuclear protein, it was named nucleoredoxin, the first member of the TRX superfamily localized into the nucleus. Further analysis revealed that NXN shuttles between the cytosol and nucleus despite the lack of the typical nuclear localization signal and nuclear export signal sequences [3,4,5]. NXN is an antioxidant enzyme that participates in the regulation of several cell processes through a redox-dependent mechanism. It performs typical disulfide reduction, necessary for the well-functioning of specific thiol proteins [6]. Several investigations have reported that NXN is a multifunctional enzyme since it functions as an oxidoreductase [3,5], is a redox-regulating enzyme, and targets reactive oxygen species (ROS) [3]. More recently, it has been reported that this enzyme has a relevant role as an oxidase enzyme by regulating the oxidative status of thiol proteins in neuronal cells [7]. Thus, NXN regulates oxidative stress but it may also function as an intermediary signaling regulator.

**Figure 1:**
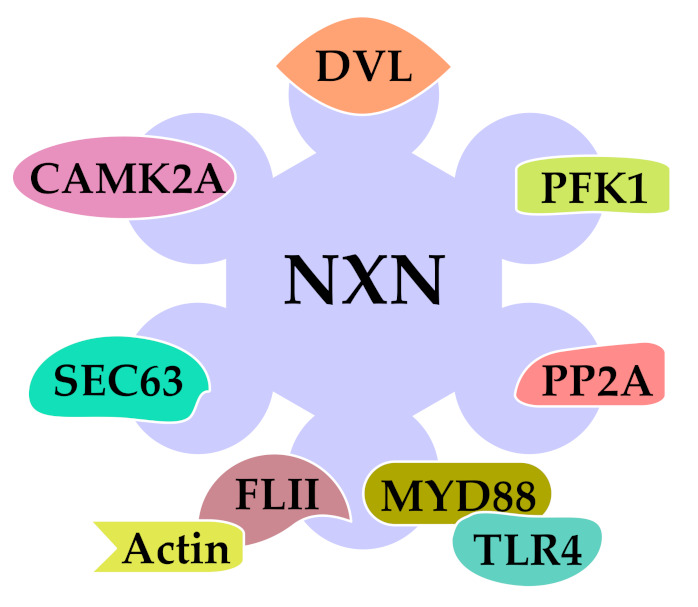
***Schematic representation of NXN redox-sensitive interactions.*** NXN directly interacts with several proteins, including DVL, PFK1, PP2A, MYD88, FLII, SEC63 and CAMK2A, which participate in the regulation of the activity of different signaling pathways. Since only some interaction domains between NXN and DVL, PP2A and SEC63 have been described so far, the image shows a hypothetical representation of the NXN-protein interactions. The interaction domains already characterized are described below.

It has been reported that NXN interacts with several proteins and contributes to cellular redox homeostasis by regulating different signaling pathways in a redox-dependent manner in animals, and targets ROS in both animals and plants. For instance, NXN regulates WNT/β-catenin pathway by interacting with DVL, a critical protein in the development and cell differentiation processes (Figure 1) [8]. It also mediates Toll-like receptor-4(TLR4)/MYD88 signaling pathway by recruiting FLII to MYD88 and regulates the nuclear translocation of the nuclear factor kappa B (NF-κB), a transcription factor that modulates innate immunity and inflammation [9]. NXN interacts with PP2A [10], suggesting that it might be involved in the regulation of protein kinase B (PKB, also known as Akt) signaling pathway since PP2A dephosphorylates PKB [11], a signaling pathway that participates in cell cycle progression, cell survival, and cell growth processes [12]. 

NXN also interacts with SEC63, a protein involved in protein transport into the endoplasmic reticulum (ER) [13]; with PFK1, a glycolytic enzyme that phosphorylates fructose-6-phosphate into fructose-1,6-bisphosphate [14,15]. PFK1 activity is regulated by fructose-2,6-bisphosphate, the product of the enzymatic activity of TP53-induced glycolysis and apoptosis regulator (TIGAR), among other enzymes, which have shown increased expression in different tumor types. PFK1 activity increases in response to proliferation signals in proliferating and cancer cells [15,16]. NXN interacts with FLII and actin, forming a ternary complex that is disrupted by the effect of chronic alcohol consumption [17]. More recently, it has been shown that NXN also interacts with and oxidases CAMK2A in mice neurons, thereby enhancing its activity, where NXN may be involved in the hyper-motivation and hyperactive behavior. It was proposed that the oxidative activity of NXN might be through the oxidation of redox-sensitive cysteine (Cys) residues of CAMK2A [18].

Based on the plethora of molecular processes where NXN is involved in, here we propose that NXN is a strong candidate to be a master redox regulator candidate of cell physiology since it regulates different redox-sensitive signaling pathways, which are implicated in the development of different pathologies. 

## 2. Oxidoreductase Activity of NXN

Although NXN belongs to a TRX subfamily, its TRX domain is slightly different from the conventional TRX domain but closely resembles that of tryparedoxin, a TRX-like protein identified in the parasite trypanosomatid [5]. However, as NXN possesses oxidoreductase activity, it has been proposed to function as a conventional TRX. Thus, if we hypothetically extrapolated the function of a conventional TRX to NXN, its oxidoreductase activity might be driven as follows. The thiol group of the N-terminal Cys residue attacks one of the sulfur atoms of Cys residues forming disulfide bonds in the substrate proteins, a so-called dithiol mechanism. Thus, the thiol group of the N-terminal Cys residue is easily deprotonated by its surrounding conditions [5,19]; therefore, it can efficiently executes a nucleophilic attack resulting in the formation of an intermediate reaction between NXN and the substrate protein that is linked by a disulfide bond [5,20].

**Figure 2:**
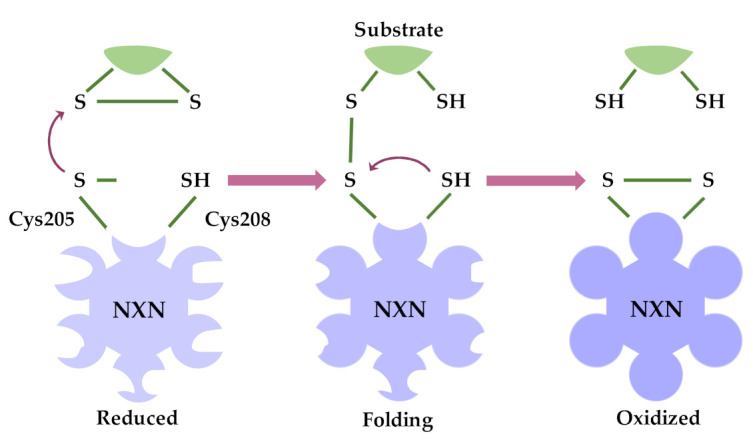
***Oxidoreductase activity of NXN.*** Based on the function of a conventional, the image shows a hypothetical function of NXN. A nucleophilic attack by the NXN Cys residue to one of the Cys residues of substrate protein initiates the reaction. NXN and the substrate protein are linked by the disulfide bond as an intermediate reaction. Then, NXN Cys208 residue attacks Cys205 residue, and the reaction ends with an oxidized NXN containing a disulfide bond, as well as, a reduced substrate protein. This mechanism might modify the conformation of NXN; however, it has been not described yet. Modified/adapted from Funato Y. and Miki H. (2007 and 2010) [3,5].

The following reaction is performed by the thiol group of the C-terminal Cys residue, which attacks the sulfur atom of the N-terminal Cys residue forming the disulfide bond in the intermediate complex, and releasing the substrate protein from NXN. A disulfide bond is then formed between the two reactive Cys residues of NXN itself (Figure 2). Then, the disulfide bond of NXN is cyclically reduced by a TRX reductase (TRXrd) by using nicotinamide adenine dinucleotide phosphate (NADPH) [5,21]. While the thiol-redox activity of NXN was demonstrated by Kurooka and coworkers [4], Funato and coworkers confirmed that the two conserved Cys residues of NXN, namely Cys205 and Cys208, are essential for its oxidoreductase activity because their mutated variants (Cys205Ser/Cys208Ser) loss such activity [8]. In part, this evidence confirms that mechanistically, the oxidoreductase activity of NXN is similar to that of conventional TRX.

## 3. Redox-Sensitive Interactions of NXN

Oxidative stress is the imbalance between oxidants and antioxidants in favor of the oxidants, leading to a disruption of redox signaling and control and/or molecular damage [22,23]. The inherent reactivity of ROS and their ability to traverse membranes pose a significant hazard that might result in common altered events, such as membrane lipid peroxidation and DNA damage [24]; therefore, ROS accumulation in the cell has been associated with a wide range of stress responses [25]. Furthermore, it has been well-established that NXN regulates several signaling pathways by interacting with different proteins, such a DVL, PP2A, PFK1, SEC63, MYD88, FLII, and CAMK2A. Although the identity of specific interaction domains between NXN and the above proteins are central have been characterized, some of them have already been identified.

NXN contains three thioredoxin-like domains, the central one comprising a catalytically active WCPPC (Trp, Cys, Pro, Pro, Cys) motif that is involved in the reduction of disulfide bonds in target proteins, and its N- and C-terminal domains, which share a high similarity to the b′ domains of protein disulfide isomerases and lack a redox active center [4]. Although several proteins have been identified as NXN-interacting, only for some of them the specific interaction domains have been characterized. 

First, it was shown that the catalytic motif of NXN is essential for regulating WNT/β-catenin pathway by binding to the basic PDZ domain of DVL and suppressing the pathway activation. It was also shown that the NXN/DVL interaction is redox-dependent in that while reducing conditions strengthen it, oxidizing conditions weaken it [8]. Then, it was shown that NXN targets the catalytic subunit either free or present in the PP2Ac-PR65/A dimer of PP2A, in NXN/PP2A interaction. Although the interaction domains identity is still unknown, it was proposed that the N-terminal domain of NXN is dispensable for such interaction [10]. Finally, the binding between NXN and SEC63 involves the C-terminal domain of NXN (amino acid residues 411–430) that is part of the third putative thioredoxin, also named b′ domain, and the Brl2 domain of SEC63 (amino acid residues 509–559) [13]. Since the protein-protein interactions are key components for their downstream functioning, the identification of the specific binding domains of both NXN and those of the interacting proteins still represents an unmet need that need to be addressed. 

### 3.1. NXN Interaction with DVL

Under physiological conditions, NXN interacts with DVL in a redox-dependent manner and negatively regulates WNT/β-catenin signaling pathway by preventing frizzled (FZD)/DVL complex formation and thereby blocking β-catenin stabilization and nuclear translocation. However, when the redox status is imbalanced, NXN becomes oxidized, leading to the dissociation of DVL from the complex, which may facilitate the phosphorylation of glycogen synthase kinase-3 beta (GSK3β) and its degradation through the proteasomal pathway [8,26]. This was first elegantly demonstrated through pull-down assays after either DVL or NXN were separately treated with hydrogen peroxide (H_2_O_2_); then, further assays revealed that NXN/DVL complex ratio decreased when NXN but not when DVL was subjected to H_2_O_2_ effects, indicating that NXN selectively responds to oxidative stress [8]. Overall, the redox-dependent regulation of different signaling pathways by NXN, can be explained by the capability of its evolutionarily conserved ROS-reactive Cys residues to sense the intracellular redox conditions [3]. Based on this concept, several redox-sensitive interactions of NXN and some of its implications on downstream signaling have been identified.

The WNT/β-catenin signaling pathway is an evolutionary conserved system that plays a crucial role in embryogenesis and organogenesis [27]. WNT signals are transduced by canonical pathway for the cell fate, and by noncanonical pathway for controlling the cell movement and tissue polarity [28]. While canonical WNT signals are transduced through FZD family receptors and LRP5/LRP6 coreceptor by activating the β-catenin signaling cascade [29,30], noncanonical WNT pathway is defined as WNT- and FZD-mediated signaling independent of β-catenin transcriptional activity [31]. WNT canonical signaling pathway is activated following the binding of a WNT ligand to FZD/LRP5/LRP6 cell surface receptor complex, recruiting DVL, which results in LRP6 phosphorylation and activation, and recruitment of Axin complex to the receptor, leading to β-catenin stabilization, which accumulates and translocates into the nucleus to form a complex with either the factor T cell factor (TCF) and/or lymphoid enhancer factor and activates WNT target gene expression. 

DVL has been largely linked to its ability to integrate and relay complex WNT signals in cells and tissues through both WNT/β-catenin and WNT/planar cell polarity (PCP) pathways, and it is regarded as the branch point between these pathways [32,33,34]. The current model of WNT/β-catenin signal transduction proposes DVL as a core protein of dynamic protein assemblies called signalosomes [35]. DVL was originally discovered in the fruit fly *Drosophila melanogaster* and three homologous namely DVL1, DVL2 and DVL3, have been identified in humans and mice. Moreover, it is well-established that DVL is a multifunctional protein that can interact with a wide range of partner proteins that either positively or negatively regulate its functioning (for details see reference [32]).

**Figure 3:**
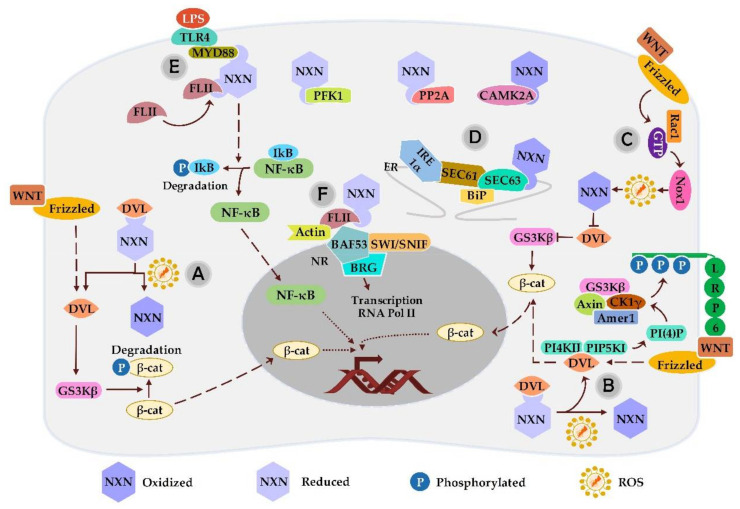
***Schematic representation of NXN-regulated signaling pathways.*** (**A**) NXN negatively regulates WNT signaling by binding to DVL, but its oxidation activates GSK3β and the downstream signaling through β-catenin stabilization and nuclear translocation. (**B**) NXN/DVL binding oxidation induces the recruitment of DVL, phosphatidylinositol 4-kinase type-IIα (PI4KII), and phosphatidylinositol-4-phosphate 5-kinase (PIP5KI) to Frizzled/WNT complex, stimulates phosphatidylinositol 4-phosphate [PI(4)P] production, and after producing phosphatidylinositol 4,5-bisphosphate [PI(4,5)P2], Amer1/Axin/GSK3β/CK1γ clustering and the subsequent phosphorylation of LRP6 is promoted. (**C**) Rac1 activation by WNT/β-catenin pathway induces generation of NOX1-derived ROS, which oxidizes and dissociates NXN from DVL. The last, suppresses GSK3β activity resulting in β-catenin stabilization and translocation into the nucleus. (**D**) NXN could be oxidizing and stabilizing the SEC63-dependent complex and contribute to co-translational protein translocation into endoplasmic reticulum (ER). (**E**) After lipopolysaccharide (LPS) stimulus, MYD88 is recruited to TLR4, which leads to IκB degradation and then, NF-κB moves into the nucleus to activate gene transcription. Upon activation of TLR4, H_2_O_2_ is produced leading to the oxidation of some NXN molecules; however, NXN also forms complex with FLII and MYD88 (FLII/NXN/MYD88) to avoid the unnecessary hyperactivation of TLR4/MYD88 signaling. (**F**) By interacting with FLII, it is likely that NXN forms a complex with nuclear receptors (NR) and participates in chromatin the remodeling process, as well as, contributes to the transcription initiation of NR-targeted genes. NXN interaction with PFK1 and PP2A could be associated with TIGAR and Akt signaling pathways, respectively; and NXN/CAMK2A interaction has not been linked yet with the participation of a specific signaling pathway, as described below.

Funato and coworkers have shown that NXN is a potent inhibitor of WNT/β-catenin signaling by interacting with DVL (Figure 3). They found that NXN overexpression selectively suppresses WNT/β-catenin pathway and the inhibition by an interference RNA (RNAi), results in TCF activation. They also showed that NXN/DVL interaction is highly sensitive to increased oxidative stress [8]. More recently, it was reported that ascorbic acid, an essential nutrient widely used as an antioxidant agent, induces non-lethal ROS levels and accelerates the release of DVL and the activation of WNT/β-catenin signaling pathway by oxidizing NXN in neural progenitor cells. The net result of this effect was improved neurogenesis by enhancing the consequent neuronal cell differentiation [36]. 

Another report has shown that treatment of colon cancer cells with WNT3A, a member of the WNT family, induces ROS production through the activation of NADPH oxidase 1 (NOX1), an enzyme responsible for the catalytic one-electron transfer of oxygen to generate superoxide or H_2_O_2_. The ROS caused by NOX1 oxidize and inactivate NXN, thereby releasing the NXN-dependent suppression of WNT/β-catenin signaling through dissociation of NXN from DVL. Authors also demonstrated that ROS induced by NOX1 is inhibited by either a specific NOX inhibitor or a NOX1 small interfering RNA; as a result, the effect of WNT3A on β-catenin stabilization and accelerated cell proliferation was also inhibited [37]. 

In *Xenopus laevis*, NXN depletion suppresses the convergent extension movements thought to underlie normal gastrulation through PCP pathway; therefore, NXN is importantly involved in the regulation of WNT/PCP pathway, where NXN inhibits DVL-induced positive regulation of c-Jun phosphorylation, probably, by activating the phosphatase activity of PP2A and promoting dephosphorylation of DVL [38]. Another scenario shows that NXN directly binds to the central PDZ domain of DVL and thus, competing with DVL-PDZ domain-binding proteins and as a result, inhibiting DVL ubiquitination and disturbing WNT/PCP pathway [5]. This scenario was corroborated by evaluating the role of NXN in preadipocyte differentiation, in which, NXN was observed to directly interact with and inhibit DVL, that subsequently allows the induction of the adipogenic transcription factor peroxisome proliferator-activated receptor gamma (PPARγ), which in turn, negatively regulates β-catenin levels, ensuring the complete terminal differentiation into mature adipocytes [39].

Another study determined that H_2_O_2_-induced oxidative stress increases WNT/β-catenin signaling and directly affects the intracellular signaling machinery in NIH3T3 cells, where NXN overexpression selectively suppresses WNT/β-catenin pathway but its ablation by a RNAi results in TCF activation, accelerated cell proliferation, and enhanced oncogenicity through cooperation with either mitogen-activated extracellular signal-regulated kinase kinase (MEK) or Ras [8]. In addition, the participation of NXN in the early progression of alcoholic liver disease (ALD) has also been investigated. The researchers demonstrated that acetaldehyde, the first ethanol metabolite, mediates β-catenin activation in a WNT-independent pathway, where acetaldehyde induces ROS production leading to NXN oxidation and the subsequent dissociation of NXN/DVL complex that induces the nuclear translocation of β-catenin, and activates fibrogenesis in human hepatic stellate cells (HSC); interestingly, NXN overexpression inhibited β-catenin nuclear translocation [26]. 

Moreover, ROS induced by chronic ethanol consumption disrupts NXN/DVL interaction in the mouse liver; as a result, phosphatidylinositol 4- phosphate [PI(4)P] production is induced following the release of DVL and formation of FZD/DVL/Phosphatidylinositol 4-kinase type-IIα (PI4KII), a complex that stimulates the synthesis of PI(4)P; once again, NXN overexpression partially reverted both the increased ROS levels and PI(4)P synthesis [40] (Figure 3). Moreover, in a similar experimental protocol, NXN overexpression inhibited the formation of FLII/β-actin complex induced by the chronic ethanol consumption and LPS treatment during ALD progression [17]. 

**Figure 4:**
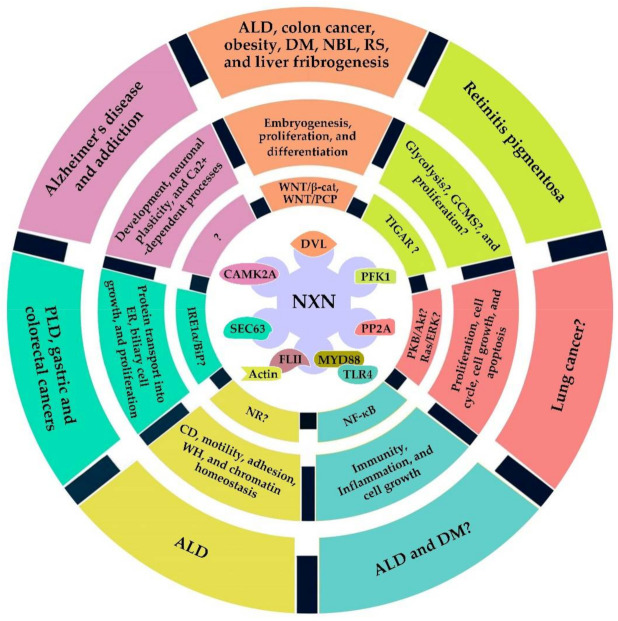
***Schematic representation of NXN interactions, involved signaling pathways, cellular processes, and associated diseases.*** NXN/DVL interaction regulates WNT signaling which influences embryogenesis, proliferation, and differentiation processes, and it has been associated with the progression of alcoholic liver disease (ALD), colon cancer, diabetes mellitus (DM), neuroblastoma (NBL), Robinow syndrome (RS), and liver fibrogenesis. NXN/PFK1 interaction could be regulating processes such as glycolysis, global cell metabolic state (GCSM), and proliferation through the participation of TP53-induced regulator of apoptosis and glycolysis (TIGAR); this interaction might be associated with retinitis pigmentosa development. NXN/PP2A interaction is likely involved in proliferation, cell cycle, cell growth, and apoptosis through PKB/Akt and Ras/Raf/MEK/ERK signaling regulation, and it could be associated with lung cancer development. FLII/NXN/MYD88 interaction regulates NF-κB signaling; as a consequence, influences immunity, inflammation, and cell growth processes; additionally, it might also be influencing cytoskeletal dynamics (CD), motility, adhesion, wound healing (WH), and chromatin homeostasis by regulating NR-dependent signaling. It is implicated in the early progression of ALD, and could be involved in DM development. NXN/SEC63 interaction is likely involved in the activation of IRE1α/BiP signaling which regulates protein transport into ER, biliary cell growth and proliferation, and it might be associated with polycystic liver disease (PLD), gastric and colorectal cancer. NXN/CAMK2A interaction could influence neuronal development, plasticity, and CA2+-dependent processes, and it is likely implicated in the development of Alzheimer’s disease (AD), and other psychiatric diseases such as addiction.

Thus, the above data indicate that NXN/DVL complex ratio is altered by different sources of oxidative stress and its dysregulation affects several cellular processes, such as organogenesis, differentiation, and proliferation, and contributes in the pathophysiology of different diseases including ALD, colon cancer, and obesity (Figure 4). Additionally, given that NXN overexpression has the capability to restore molecular alteration induced by increased ROS production, this evidence also suggests that NXN manipulation is an attractive therapeutic strategy that might be explored during the progression of diseases where NXN has been involved.

### 3.2. NXN Interaction with PFK1

PFK1 is the second rate-limiting enzyme involved in glycolysis. It is widely held to dictate the pace of glycolytic flux [41] and is primarily synthesized as an unstable and inactive monomer, which can rapidly form dimers [42]. However, its active form is tetrameric and the formation and stabilization of PFK1 tetramers largely influence the glycolytic flux rate [43]. The requirement of increased glycolysis by neoplastic cells has suggested that rate-limiting enzymes, such as PFK1, may serve as an essential control point during neoplastic transformation. This is supported by the fact that many glycolytic enzymes including PFK-1 are commonly elevated, leading to elevated glycolytic flux, which promotes the proliferation of cancer cells [44,45,46]. Moreover, fructose-2,6-bisphosphate (F-2,6-BP), a product of fructose-6-phosphate, which is catalyzed by fructose-2,6-bisphosphatase-3, has been considered the most potent allosteric activator of PFK1, and may increase PFK1 activity even in presence of ATP [43]. In contrast, increased expression of TIGAR diminishes F-2,6-BP levels, resulting in an inhibition of glycolysis [47]. 

In addition, it has been shown that human bone osteosarcoma epithelial cells (U2OS cell line) expressing TIGAR have higher NADPH levels and a concomitant enhanced ability to regulate ROS levels and thus reduce oxidative stress in the cell [47,48]. Also, it has been proposed that TIGAR expression would be detrimental for cells that are highly dependent on glycolysis either for survival or under conditions where the promotion of glycolysis contributes to tumorigenesis [47]; however, unregulated expression of TIGAR may also help aberrantly proliferating tumor cells to survive toxic levels of oxidative stress [47,49].

Recently, using mouse embryonic fibroblasts (MEF) lacking the *Nxn* gene, it was shown that NXN is a novel regulator of both oligomerization and PFK1 catalytic activity. NXN deficiency increased NADPH levels and reduced glutathione, two of the major cellular antioxidants generated through the pentose phosphate pathway (PPP); as a result, it makes cells more resistant to oxidative stress. However, the exact mechanism by which NXN might affect PFK1 oligomerization remains unclear since PFK1 is also subject to glycosylation, phosphorylation, and acylation [14]. Finally, based on the role of PFK1 as a key enzyme in the regulation of the global cell metabolic state (GCMS), as well as, on the fact that PFK1 alteration contributes to the development of multiple human diseases (Figure 4) [50], its interaction with NXN strongly suggests that dysregulation of PFK1-dependent functions might be closely linked to the chronic presence of increased oxidative stress that persistently oxidizes NXN.

### 3.3. NXN Interaction with PP2A

One of the most versatile and essential phosphatases involved in cell division is PP2A. This phosphatase regulates every stage of the cell cycle in several critical pathways and, not surprisingly, it has been widely implicated in the tumor suppression process [51]. PP2A is a heterotrimeric enzyme composed of a scaffolding subunit A (PP2A-A), a regulatory B subunit, and catalytic subunit C (PP2A-C). The A and C subunits form the core enzyme, which interacts with a B-subunit to create a holoenzyme [52]. PP2A must be activated before being assembled into active holoenzymes, by phosphotyrosyl phosphatase activator (PTPA), also known as PP2A-specific phosphatase activator. PP2A and PTPA forms a combined ATP-binding pocket to directly chelate catalytic metal ions, subsequently, the phosphatase active site catalyzes ATP hydrolysis. Finally, this process becomes crucial for efficient loading of authentic catalytic metal ions and acquisition of pSer/Thr-specific phosphatase activity [53].

PP2A appears to have a primary role in regulating mitogen-activated protein kinase pathway [54]. PP2A can bind to the phospho-tyrosine binding domain of SRC homology-2-containing protein (SHC), an essential member of the complex that binds growth receptors, and negatively regulates Ras activation [55]. It has also been observed that it can positively regulate that pathway through the protein complex composed by SHC, growth factor receptor-bound protein 2, and son of sevenless protein. This complex activates Ras which starts a signal cascade from the activation of Raf, to activate MEK, ERK, and eventually the transcription factors that trigger the transcription of growth-related genes [54,56] (Figure 4). PP2A can indirectly regulate the induction of apoptosis via dephosphorylation of the cell signaling [54,57,58], and directly act on both apoptotic and anti-apoptotic proteins [59].

PKB/Akt, is a serine/threonine-specific protein kinase that plays a key role in glucose metabolism, apoptosis, cell proliferation, and cell migration, and it is mainly regulated by protein kinases and phosphatases [60,61]; both are highly expressed in the kidney, spleen, and liver. In the latter, it has been shown that PP2Aregulates Akt activity. Overexpressed-eukaryotic translation initiation factor 3 subunit I interacts with the activated form of oncogenic Akt1 via inhibition of PP2A phosphorylation [62], whereas the protein regulated in development and DNA damage responses 1 enhances PP2A-mediated dephosphorylation of Akt, resulting in the repression of mammalian target of rapamycin complex 1 signaling in human embryonic kidney cells [63]. PP2A inactivates Akt by dephosphorylation but the reduced expression of PP2A B55 alpha subunit results in increased activation of Akt and consequently, increased cell proliferation and growth of lung tumors [64].

Recently, it has been shown that PP2A interacts with the N-terminal region of NXN, where the target of NXN is the catalytic subunit either free or forming the PP2Ac-PR65/A dimer, and Cys 269 and 272 could be the direct targets for NXN-catalyzed disulfide bond formation [10]. It should be taken into account that reactive nitrogen species (RNS) and ROS, indirectly and directly, respectively, regulate the activity of PP2A and they have key implications in cancer proteome and in the assembly/activity of PP2A holoenzyme, respectively [65]. Interestingly, it has been described that TRXR1, a member of the TRX family, directly protects the protein tyrosine phosphatase 1B, from inactivation in oxidizing microenvironments [66]. 

Therefore, it is plausible to propose that in PP2A/NXN interaction, NXN might be protecting PP2A from the adverse effects of oxidative stress and free radical accumulation, an NXN-dependent function that might influence the regulation of PP2A-dependent functions, such as that on cell cycle, proliferation, apoptosis, among other processes. This represents an intriguing hypothesis that should be clarified.

### 3.4. NXN Interaction with MYD88, FLII and Actin

MYD88 was first described as a gene upregulated during IL-6-induced myeloid differentiation [67]; interestingly, then it was shown that MYD88-deficient mice do not respond to lipopolysaccharide (LPS), the ligand of TLR4 and a key component for the downstream inflammatory signaling [68]. Thus, MYD88 has been considered as a hub in inflammatory responses and its dependent signaling can lead to the production of either pro- or anti-inflammatory cytokines [69], ). For example, when the immune response is initiated through TLRs, proinflammatory cytokines are produced by the activation of several downstream proteins and transcription factors, such as NF-κB [70]. NF-κB regulates the expression of both immune and growth genes, which can be activated through either the classical or alternative pathway [71,72], and TLRs play a central role in innate immunity mediated by NF-κB [73]. 

TLRs are an important family that recognizes conserved microbial molecules and activates pivotal signaling pathways for the innate and adaptive immune responses [74]. Immediately downstream of most TLRs is MYD88, an essential adapter protein that functions as a signal transducer [75]. TLR4 is one of the most important and key TLRs that contains a well-known Toll/IL-1 receptor domain in its cytoplasmic region, which binds to the carboxyl end of the intracytoplasmic adaptor protein MYD88 to form a complex; then, this complex binds to IL-1 receptor-related kinase and phosphorylates itself. After binding to tumor necrosis factor receptor-related factor 6, IκB kinase is activated, causing ubiquitination and degradation of IKB to activate NF-κB, which is translocated from cytoplasm to the nucleus to either initiates or enhances the transcription several genes [76,77]. 

Another protein that also interact with MYD88 and play an important role in TLR-mediated signaling is FLII [78,79].FLII is a multifunctional protein and recently has been identified as an emerging regulator of inflammation [80]. The leucine-rich region of FLII shares 29% sequence identity and 42% similarity to TLR4, suggesting that FLII may influence TLR signaling [81]. In 2006, human FLII homolog (FLIIh) was first characterized as a negative regulator of NF-κB activity by interfering with TLR4/MYD88 interaction [81]. More recently, it was reported that NXN might share a common function as suppressors of the TLR4/MYD88-dependent signaling since NXN is a required adapter protein to recruit FLII to MYD88 (Figure 3). Authors showed that after LPS exposure of MEF cell culture, MYD88 is recruited to TLR4, which leads NF-κB activation downstream; however, in NXN-deficient cells, FLII cannot hijack MYD88 from TLR4 and thus, TLR4/MYD88 signaling is hyperactivated upon LPS stimulation. Therefore, NXN interacts with FLII and MYD88 to negatively regulate and avoid the unnecessary activation of TLR4/MYD88 signaling [9]. 

Under normal conditions, FLII acts as a cofactor and plays a central role on actin remodeling by interacting with actin-based structures, among other functions [82]. Recently, it was shown that NXN forms a ternary complex with FLII/Actin complex that is differentially disrupted by ROS produced in ALD in vitro and in vivo models. Based on the role of the FLII/Actin complex, it was suggested that by interacting with this complex, NXN could be participating in the regulation of motility, contraction, adhesion and wound healing processes, that might be altered during ALD progression (Figure 3 and Figure 4) [17]. In this line, recently it has been proposed that through its oxidase activity, NXN may inhibit Cofilin 1, a regulator protein of F- and G-actin polymerization, and negatively regulate cytoskeletal dynamics and motility in cells from neuronal origin (Figure 4) [7].

FLII not only binds to actin but also to actin-related proteins including SWItch/Sucrose Non-Fermentable (SWI/SNF) related, matrix associated, actin-dependent regulator of chromatin, Subfamily A, Member 4 (SMARCA) known as BRG1-associated factor 53 (BAF53) [83]. Both actin and BAF53 are key components of the SWI/SNF chromatin remodeling complex, which is required for the transcription initiation of nuclear receptor (NR)-targeted genes. In this process, SWI/SNF complex incorporates two molecules of either actin, BAF53, or one of each at the promoter site [84]; in addition, it is also well-known that FLII is required for the maintenance of optimal chromatin configuration at the enhancers of estrogen target genes, to facilitate the binding of RNA polymerase II to the promoter region of the target gene (Figure 3) [85]. 

The above evidence suggests that NXN may be involved in the regulation of immunity and inflammation by interacting with FLII and MYD88 upstream of NF-κB transcription factor, leading to the production of both pro- and anti-inflammatory cytokines; moreover, it strongly suggests that NXN may also participate in the regulation of cytoskeletal dynamics and cell motility, contraction, adhesion, and wound healing, as well as in the chromatin homeostasis.

### 3.5. NXN Interaction with SEC63

SEC63 has been identified as a critical factor that deactivates inositol-requiring enzyme 1 alpha (IRE1α) activity during persistent ER stress [86]. It is well-known that IRE1α is the most ancient ER stress sensor, conveying a critical signaling response through its RNase activity [87]. During ER stress, SEC63 recruits and activates BiP ATPase via its luminal J-domain to bind onto IRE1α, thus suppressing the RNase activity of IRE1α [86]. SEC63 resides in the ER membrane and is involved in co-translational protein translocation into the mammalian ER (Figure 3) [88,89]. It consists of three transmembrane domains and the most important is known as J-domain which allows the interaction with chaperone proteins, such as binding immunoglobulin protein, to facilitate the unidirectional translocation of precursor proteins through the SEC61 translocation pore [89], thus, when the protein reaches the ER membrane, a nascent chain engages the trimeric SEC61 complex [90] to form a larger protein complex with the SEC62/63 complex, so-called SEC complex [91]. Then, this complex is associated with the SEC61 complex for the translocation of selective small-size substrates, namely, less than 160 amino acids [92].

The interaction of SEC63 with NXN was first observed in a yeast two-hybrid screen involving the carboxy-terminus of NXN and the Brl (Brr2-like) domain in the COOH-terminal cytosolic region of SEC63. Interestingly, it was shown that this interaction is also redox-sensitive, but appositively to NXN/DVL interaction, NXN/SEC63 interaction was stimulated under oxidizing conditions [13,91]. Based on this evidence, authors proposed that oxidative stress favors WNT/β-catenin signaling by simultaneously inhibiting NXN/DVL but stimulating NRX/SEC63 interaction; as a result, both events suppress the formation of NXN/DVL complex. They also hypothesized that the possible scenario for this phenomenon is that the cytosolic domain of SEC63 provides a binding platform for NXN, which affects the NXN availability to interact with DVL [13]. 

Altogether, these data show that by interacting with SEC63, NXN might be stabilizing SEC63 functioning; thus, it is involved in the regulation of protein transport into the ER, an essential cellular process that depends on SEC63, but when this protein is dysfunctional, it may contribute to some pathologies associated to the protein transport into the ER, as evidenced below.

### 3.6. NXN Interaction with CAMK2A

CAMK2A is a serine/threonine kinase that is highly abundant in the brain, especially in the postsynaptic density [93]. The major neuronal CAMK2A subunit contains a catalytic kinase domain and a Ca2+/calmodulin-binding regulatory domain containing modulatory autophosphorylation sites [94,95]. This protein has numerous roles in mediating cellular responses through the regulation of intracellular Ca2+ (Figure 4), such as, alterations in neurotransmitter synthesis, ion channel regulation, cell division, modulation of muscle contractility, and gene transcription [96]. CAMK2 has been found in most tissues but in neurons, it is in high concentrations since may be up to 2% of total protein in some brain regions [97,98]. Its activity can be modulated at many levels but autophosphorylation at threonine 286 is mandatory for the major forms of synaptic plasticity in the hippocampus [99,100].

Recently, it has been identified CAMK2A as a strong interaction partner of NXN [18]. By using a yeast-2-hybrid screen and analysis of differentially oxidized proteome, as well as a NXN knockout mice model that partially deletes NXN functioning; i.e., while *NXN* mRNA was reduced by up to 90%, its protein level was only partially reduced, the authors found reduced oxidation in hippocampus neurons, including CAMK2A oxidation in mice. The loss of NXN-dependent pro-oxidative functions was manifested as a significant reduction of non-goal-directed behavior, restricted efforts to goal-directed behavior with lower interest in adventurous, exploratory and rewarding activities including voluntary wheel running. Since exploration in mice depends on CAMK2A and in turn, the activity of this enzyme is strongly influenced by NXN, the authors rightly proposed that NXN may sustain motivation and pleasure on exploration, likely, by maintaining CAMK2A oxidation and activity [18]. 

A more recent neurological study that investigated the link between *Nxn-like 2* (*Nxnl2*), a paralog gene of *Nxn*, and tauopathies, in young and old mice. Results showed that young *Nxnl2*−/− mice had severe behavioral deficiency in fear, pain sensitivity, coordination, learning and memory, as well as deficits in long-term potentiation, which revealed that this gene is playing a key role in regulating brain functions. Additionally, glucose metabolism in the hippocampus was also reduced but it was not corrected by gene therapy. In old mice, *Nxnl2−/−* phenotype showed brain stigmas of tauopathy, such as oligomerization, phosphorylation and aggregation of TAU. Thus, while *Nxnl2−/−* young mice resemble mild-cognitive impairment, *Nxnl2−/−* old mice exhibit tauopathy, a clinical condition closely associated to Alzheimer’s disease progression [101].

Together, the above data strongly suggested that NXN maintains the oxidative state of CAMK2A and thereby its activity, which was supported by previous findings showing that NXN mainly acts as an oxidase enzyme in neuroblastoma cells [7]. Therefore, NXN/CAMK2A interaction and loss of CAMK2A oxidation upon NXN deletion suggest that mice behavioral manifestations involve redox modification of CAMK2A, and are contributed by further NXN-dependent protein oxidations of synaptic and mitochondrial proteins [18]. Thus, NXN-dependent oxidation of CAMK2A plays a central role in the proper functioning of the brain. Moreover, the data also support the notion that a paralog of *Nxn* gene, namely *Nxnl2*, also contributes to the brain well-functioning [101]. Thus, their synergistic participation in the progression of brain diseases represents an intriguing hypothesis that need to addressed.

## 4. NXN Implication in Pathologies

The above data describe that NXN interactions influences the downstream functioning of several redox-sensitive signaling pathways, either through its reductase or oxidase activity, that in turn, may modify the associated cellular processes. The involvement of NXN in the homeostasis of a broad spectrum of key cellular processes implies that its redox-regulated processes may be also affected and contribute to the establishment of different diseases. Table 1 shows the summarized information on the implication of NXN in several diseases.

### 4.1. Diabetes Mellitus

Diabetes mellitus (DM) is a group of metabolic disorders characterized by hyperglycemia and insufficiency of either production or function of insulin [109]. DM is mainly classified as type I (insulin-dependent) due to immune-mediated beta cells destruction, leading to insulin deficiency; and type II (non-insulin-dependent) due to insulin-secreting defect and insulin resistance. However, this condition might also be idiopathic or gestational [110]. Insulin is a hormone synthesized by beta cells in the pancreas in response to various stimuli. Long-term elevation of glucose levels in the blood is associated with complications leading to heart disease, stroke, blindness and kidney disease. Several factors play important roles in the pathogenesis of DM, such as hyperlipidemia and oxidative stress [109]. In type I DM, oxidative stress participates in beta cell destruction and beta cell failure due to chronic hyperglycemia leading to toxicity in type II DM. In an in vivo study where the type I was induced with streptozotocin (STZ) administration, overexpression of TRX in beta cells was found to prevent STZ-induced DM. In this study, antioxidant treatment was reported to prevent beta cell dysfunction in a type II DM mice model. 

Because *Nxn* gene localized on chromosome 11 is a positional and functional candidate for STZ sensitivity, *Nxn* sequence was analyzed in three DM-associated mouse models. Authors used a nonobese diabetic mouse recapitulating type I DM, the Nagoya–Shibata–Yasuda mice model, which recapitulates type II DM, and an STZ-resistant *Nramp* wild-type mouse model. Results showed that *Nxn* mutations might be involved in the pathogenesis of STZ-induced DM and in that of type I and type II DM. Authors proposed that *Nxn* mutations might sensitize beta cells to oxidative stress-induced damage. Therefore, it was suggested that *Nxn* might be an important candidate gene for DM development [111]. Unfortunately, the effects of *Nxn* mutations on the status of NXN-dependent interaction was not investigated; however, it is well-documented that NF-κB plays a key role in the pathogenesis of vascular complications of DM since persistent hyperglycemia induces oxidative stress and activates NF-κB triggering the expression of various cytokines, chemokines, among other molecules [112]. Based on this rationale, it is plausible to speculate that *Nxn* mutations induced by these DM models may be altering the status of FLII/NXN/MYD88 interaction which regulates downstream NF-κB activation (Figure 3 and Figure 4). However, this approach still needs to be addressed.

### 4.2. Obesity

The World Health Organization defines obesity as abnormal or excessive fat accumulation representing an important health risk worldwide [113]. A recent study has reported that obesity prevalence significantly increased between 1975 and 2016 worldwide. Obesity is a major risk factor for several chronic diseases, including metabolic disorders (DM and fatty liver disease), cardiovascular and musculoskeletal diseases, as well as some types of cancer and as a result, it might lead to reduced quality of life. The long-term energy imbalance between excessive consumption and low expenditure of calories is the main cause of obesity [114]. 

Adipose tissue is key in the regulation of overall-body energy homeostasis. Excessive calories consumption increases both size and number of fat cells. Obese adipose tissue promotes mild chronic inflammation and insulin resistance, which result in severe obesity and DM [115,116]. Adipogenesis is controlled by a balance of internal and external factors that either stimulate or repress adipogenic differentiation. In the early phase of adipogenic differentiation, CCAAT/enhancer-binding protein alpha (C/EBPα) and PPARγ control the differentiation of preadipocytes into lipid-accumulating fat cells. PPARγ suppress canonical WNT signaling by proteasome-dependent degradation of β-catenin. In turn, β-catenin, interrupts adipogenesis by repressing PPARγ and C/EBPα [117]. Of note, several reports have suggested a close relationship between WNT/β-catenin signaling, DM development, and adipogenesis [117,118,119].

Recently, it has been described that NXN modulates adipogenic differentiation through the regulation of WNT/β-catenin signaling. Results showed that *Nxn* mRNA and protein were increased in the early stages of adipocyte differentiation in white adipose tissue of a leptin-deficiency model of obesity (ob/ob mice). Based on experiments performed in both *Nxn*-depleted and *Nxn*-overexpressing 3T3-L1 preadipocytes, it was shown that NXN participates in the adipogenic differentiation of these cells. In addition, differentiation of primary adipocytes from adipose tissue-specific *Nxn* transgenic (Adipo-*Nxn*) mice was increased in vitro, and the epididymal and perirenal fat was increased in Adipo-*Nxn* mice in vivo. Adipo-*Nxn* mice exhibited hyperplasia and hypertrophy in adipocytes, decreased expression of enzymes involved in lipolysis and fatty acid oxidation [39]. Interestingly, it has been shown that hypertrophic adipocyte is likely caused by decreased triacylglycerol catabolism, a condition associated with the occurrence of other diseases such as obesity and type 2 DM [120]. 

Adipo-*Nxn* mice also showed a trend toward glucose intolerance and mild insulin resistance, besides an increased expression of inflammatory and macrophage markers [39]; in this line, obesity-induced insulin resistance strongly correlates with increased infiltration of inflammatory cells in adipose tissue [121]. Authors found that NXN increased its interaction with DVL and inhibited the activation of WNT/β-catenin signaling during adipogenesis. They also showed that the negative regulation of WNT/β-catenin signaling by NXN induces adipogenic transcription factor *PPARγ* expression, which downregulates β-catenin levels and promotes terminal differentiation into mature adipocytes [39]. This evidence indicates that NXN contributes to obesity control (Figure 4) by acting as a proadipogenic factor, placing NXN as an attractive therapeutic target in obesity and metabolic disorders such as DM.

### 4.3. Brain Diseases

Alzheimer’s disease (AD) is a neurodegenerative disorder characterized by a progressive decline in cognitive functions, including memory, thinking, language, and learning capability. The pathophysiology of AD involves the downregulation of neuronal functions and upregulation of innate immune responses in AD brains [122]. In AD, the injury and death of neurons start in the hippocampus brain region, and then atrophy affects the whole brain. This disorder is defined by the accumulation of toxic senile amyloid plaques (neuritic plaques) and neurofibrillary tangles in the brain. These deposits are composed of misfolded protein aggregates, made up mainly of amyloid beta-peptide and Tau protein. Sometimes, these aggregates are deposited in the walls of small blood vessels in the brain, a process known as amyloid angiopathy [123].

Recently, it has been shown that the inactivation of *Nxnl2* gene induces stress and Tau hyperphosphorylation. Microarray analysis of retinal RNA obtained from an *Nxnl2−/−* mouse model showed that the transcription factor Sox30 and Transgelin 2 were upregulated. Transgelin 2 encodes a protein involved in the organization and dynamics of actin cytoskeleton [124]. Interestingly, Transgelin 2 is overexpressed in the brains of AD patients [125]. Furthermore, it was observed that Tau protein is hyperphosphorylated in the retina of *Nxnl2-/-* mouse model, a phenomenon observed in neurofibrillary tangles of AD patients [124], which suggests that NXN might be involved in the development of AD.

The human neuroblastoma SH-SY5Y cell line has been a useful cellular model for the study of neuroblastoma (NBL), neuronal aging, and neurodegenerative diseases such as AD [126]. NBL is the most common extracranial solid tumor in children, accounting for 7% of all pediatric neoplasms in patients under 15 years old and 15% of all pediatric deaths caused by cancers. It is the second most common type of pediatric cancer and the world mortality rates are 0.85–1.1 cases per 100,000 children under 15 years-old [127]. A recent redox proteomic analysis performed in SH-SY5Y cells revealed that NXN deletion mostly resulted in a higher fraction of reduced proteins, suggesting that NXN acted primarily as oxidase for thiol proteins, which were mainly involved in development and cell morphology processes [7]. 

In addition, it has been observed that *NXN* knockdown in SH-SY5Y cells, antagonism, or pharmacologic inhibition might be able to fight aging since *NXN* knockdown generated a high rate of self-renewal, autophagy, and upregulation of redox-sensitive heat shock proteins, such as Hsc70/HSPA8 and HSP90. Since in neuronal cells the enhancement of autophagy and maintenance of high neurogenesis have been proposed as anti-aging mechanisms, authors anticipated that if the inhibition of NXN in vivo were possible, it might be a valuable anti-aging strategy, a proposal that has to be carefully addressed because NXN inhibition might also foster cancer growth [102]. 

Although the above reports closely associate the role of NXN in neuronal defects, no one has investigated the redox-interacting capability of NXN in order to determine whether a signaling pathway regulated by NXN was involved. Interestingly, a recent investigation demonstrated that NXN interacts with and sustains the activity of CAMK2A [18], a postsynaptic kinase that is crucial for neuronal plasticity [128]. Neuronal plasticity is a fundamental process allowing the brain to receive information and generate appropriate adaptive responses to different stimuli, including environmental, social, behavioral, and pharmacological. Moreover, the activity of CAMK2A has also been implicated in several psychiatric diseases, including autism, schizophrenia, and addiction (Figure 4) [129,130]. Thus, although a signaling pathway linking the altered cellular processes in neurons and brain diseases with NXN involvement has not yet been determined, a tangible possibility is that a modification of NXN/CAMK2A interaction ratio induced by oxidative stress could initiate the molecular mechanism that eventually will affect neuronal processes such as, development, morphology, and plasticity, promoting different brain diseases.

### 4.4. Hepatic Diseases

ALD is one of the major causes of morbidity and mortality worldwide and its clinical spectrum includes steatosis, fibrosis, alcoholic hepatitis, cirrhosis, and hepatocellular carcinoma (HCC) [131]. The disease can be caused by the chronic consumption of alcohol exceeding a certain daily amount [132], which is oxidized by alcohol dehydrogenase to acetaldehyde in hepatocytes [133]. Acetaldehyde production increases oxidative stress, which is mediated by ROS; in turn, ROS bind directly to and damage DNA, and lead to lipid peroxidation generating lipid peroxidation products, such as 4-hydroxynonenal and malondialdehyde [134]. When ROS levels exceed the capability of the endogenous antioxidants, cells are exposed to oxidative stress which causes severe dysfunctions or cell death; therefore, redox balance plays a critical role in the alcohol-mediated cellular fate [135].

Recently, it has been shown that acetaldehyde, the first metabolite of ethanol oxidation, mediates β-catenin in a WNT-independent pathway, through the imbalance of NXN/DVL interaction ratio that in turn induces β-catenin nuclear translocation and activates fibrogenesis in human HSC [26]. This interaction also participates in the regulation of PI(4)P production but during ALD progression, oxidative stress induced by ethanol consumption disrupts the homeostatic NXN/DVL interaction ratio and stimulates FZD/DVL/PI4KII complex formation and as a result, the production of PI(4)P is increased both in vivo and in vitro [40]. 

Besides, by using the same ALD models, authors also demonstrated that chronic ethanol consumption disrupts FLII/NXN/MYD88 complex stimulating the release of FLII into the bloodstream and culture media, respectively; thus, they proposed FLII as a non-invasive biomarker for detecting the early ALD progression [136]. Together, this evidence indicates that some NXN-dependent interactions are sensitive to the oxidative stress produced by ethanol metabolism, and strongly suggests that NXN may be a key player during ALD progression.

In 2004, SEC63 was the first human SEC protein linked to a human disease [88] by showing that the loss of SEC63 may cause changes in the ER homeostatic microenvironment disrupting the precise folding of polycystin-1 [137]. Further studies showed that the role of *Sec63* as a driver gene in the pathogenesis of the autosomal-dominant polycystic liver disease (PLD) is associated with the disruption of co-translational protein transport [138,139]. Although the mechanisms of PLD development are not well-established, it has been proposed that proteins such as SEC63, are essential for the biogenesis of either a single or a set of proteins regulating the biliary cell growth and proliferation, but in the absence of SEC63 function, that set of proteins do not reach their functional location, which could result in a proliferative advantage for the progeny of the respective cells [13]. 

Besides, both mutations and overexpression of *Sec* genes have frequently been associated with various human cancers. For instance, frameshift mutations in *Sec63* gene, caused by microsatellite instability, have been found in 37.5% of microsatellite-unstable gastric cancers, 48.8% of colorectal cancers [107], and in one case of HCC associated with Lynch syndrome [108]. Thus, by interacting with SEC63, NXN might also contribute to the progression of some diseases such as PLD and different cancer types (Figure 4).

### 4.5. Retinitis Pigmentosa

Inherited retinal degenerations (IRD) are heterogeneous disorders that have recently become critical clinical targets for gene therapy [140]. Retinitis pigmentosa (RP) or hereditary retinal dystrophy refers to several disorders promoting the gradual vision loss. This condition affects approximately one in 5000 people worldwide, making RP the most common inherited disease of the retina [141]. Currently, several genetic mutations related to this disease have been revealed. One of the most important is the dominant rhodopsin gene mutation, where around 150 mutations have been found [140]. For example, *RPE65* gene is essential for the trans-isomerization of all-*trans*-retinol to 11-*cis*-retinal retinal esters [141]; however, the absence of conversion of these esters interrupts the functional resupply of light-sensitive opsin proteins, located in both rods and cones photoreceptor cells [142]. 

RP is characterized by progressive damage from night blindness, originated by the death of rods, culminating in complete blindness due to cones dysfunction located in the retina center, specifically in the fovea. RP is associated with a complete loss of rods in advanced stages, with some remaining foveal cones characterized by shortened and disorganized outer segments [140]. In normal conditions, phototransduction begins in the photoreceptors outer segment, these are composed of enriched lipid bilayers stacks with poly-unsaturated fatty acids, prone to oxidation, and in this place, opsin proteins are located [143].

Recent findings have proposed that loss of rods triggers a reduction in the rod-derived cone viability factor (RdCVF) expression, a splicing variant of the NXN-like-1 (*Nxnl1*) gene. Interestingly, TRX RdCVF expression is affected in both rods and cones of mice carrying homologous recombination of *Nxnl1* gene. This condition leads to further oxidative damage and progresses with age. Mice bearing this deficiency have a higher concentration of adducts produced by lipid peroxidation. *Nxnl1* participates in the restoration of the glycolytic enzymes function due to reduction of oxidized thiol groups of Cys. Aerobic glycolysis is necessary for rod outer segment renewal, a parallel phenomenon to that described for cones. Metabolic and redox signaling disruption, between rods and cones by the loss of RdCVF expression, reduces cone vision due to its shortening outer segments. RdCVF specifically interacts with a complex formed by single-pass type 1 transmembrane domain basigin protein and the glucose transporter 1 at cones surface. RdCVF stimulates aerobic glycolysis and provides necessary triglycerides for cones outer segments renewal. RdCVFL reduced power (second *Nxnl1* gene-splicing variant) relies on the glucose metabolism by cones whose absorption is stimulated by RdCVF. RdCVFL must be reduced by TRXrd, which requires NADPH cofactor reduced form. Glycolysis inhibition by ROS leads to glucose-6-phosphate accumulation, which is redirected to PPP, producing two NADPH molecules necessary for TRXrd enzyme activation [104,144].

To determine the molecular and clinical aspects involved in different IRDs types, murine RP models such as rd1 and rd10 RP have been developed [145,146,147,148]. Thus, the role of RdCVF was determined in the mouse retina by using phenotype analysis of mice bearing altered *Nxnl1* gene. Authors showed that the downregulation of RdCVF leads to secondary degeneration of cones resulting in the RP development [104,149] This evidence indicates that the functioning of the NXN homologous, namely *Nxnl1*, is central in maintaining the integrity of cones and in turn, the homeostatic vision. An intriguingly hypothesis is that if in addition to mutations, the alteration of some redox-sensitive interactions of NXN also contributes in the progressive vision loss associated with RP.

### 4.6. Robinow Syndrome

Skeletal dysplasia forms a complex condition with extraordinary molecular and clinical heterogeneity, such as Robinow syndrome, the least frequent Robinow syndrome (RS) form [150], a disease particularly frequent in Turkey [151]. RS patients are characterized by short-limb dwarfism, costovertebral segmentation defects, abnormal head, face, and external genitals morphogenesis. Patients may also have brachydactyly, hands polydactyly, feet oligodactyly, as well as cardiac malformations [103,151]. The relevance of heterozygous mutation in receptor tyrosine kinas- like orphan receptor 2 (*ROR2*), a gene involved in the early formation of the chondrocytes, cartilage and growth plate development, in the development of the most severe clinical form of RS has been previously described [152]. 

Furthermore, a close link between RS and WNT signaling has been reported since all currently known pathogenic variants of RS, including *ROR2*, *WNT5A DVL1,* and *DVL3*, are related to non-canonical WNT signaling. This pathway establishes cellular orientation via WNT/PCP pathways. Additionally, the mutagenic variant encoding *FZD2* gene and the biallelic variant encoding *Nxn* gene have also been described, and their protein products are relevant partners in the interactome of WNT5A, playing key roles in skeletal development. It has also been shown that *Nxn* gene is highly expressed during mice extremities development [103].

Copy number variants (CNV) elimination may explain mutations in some alleles associated with RS, such as the specific *NXN* exon 1 CNV deletion [153]. Interestingly, the abnormal WNT/β-catenin signaling activation in *Nxn-/-* knockout mice leads to craniofacial defects, a relevant phenotype in subjects with RS, likely due to the loss of function of *NXN* biallelic variants [103]. As already mentioned, NXN acts as a negative regulator of WNT/PCP pathways in both cell culture and animal models. Signaling disruption to one or both pathways could lead to the craniofacial abnormalities observed in *Nxn* mutation models. For example, a mutation in chromosome 11 that alters a consensus splice site of *Nxn* (*Nxn^J13/J13^*) and inserts 10 amino acids into the resulting protein, kills 97% of *Nxn^J13/J13^* mutant mice on postnatal day one. Moreover, embryos present craniofacial dysmorphology, cleft palate and small jaw, and abnormal craniofacial morphology partially recapitulates the phenotype observed in RS patients [154]. 

In a study performed in *Xenopus laevis* embryos, where fertilized eggs at four or eight cell stage were injected with either *Nxn* mRNA to overexpress or Nxn morpholino antisense-oligonucleotides to deplete *Nxn* gene expression, authors demonstrated that endogenous *Nxn* expression functions as a negative regulator of WNT/β-catenin pathway signaling where NXN protein bound to DVL in a redox-dependent manner [8]. A different study showed that *Nxn* overexpression or depletion produces the bent-axis phenotype, typically observed in embryos with abnormal PCP pathway activity. Thus, NXN acts as a potential negative regulator of WNT/PCP pathway by blocking and inhibiting DVL-induced up-regulation of c-Jun phosphorylation through Rac, a crucial molecular mechanism regulating WNT/PCP pathways [38]. Together, these data suggest that RS, in part, results from WNT/β-catenin and/or PCP pathways disturbance during human development, two signaling pathways negatively regulated by NXN, and show the relevance of *Nxn* biallelic variant in the onset of RS recessive form in rodents (Figure 4).

## 5. Conclusions and Future Directions

The oxidoreductase and redox-dependent interaction capabilities, have placed NXN as an enzyme capable of participating in different cellular processes regulated by redox stimuli, such as ROS, a critical component in life evolution since it exerts a broad spectrum of biological effects, ranging from physiological regulatory functions to molecular alterations that contribute to the pathogenesis of various diseases. While through its oxidoreductase capability NXN protects the activity of different enzymes, such as that of catalase and CAMK2A, through its redox-sensitive interactions regulates the downstream activity of several signaling pathways, including WNT/β-catenin and TLR4/MYD88/NF-κB. 

An intriguing phenomenon is that the alterations of some molecular mechanisms, such as increased nuclear translocation of β-catenin, liver fibrogenesis activation, ROS production, PI(4)P synthesis, and the formation of FLII/β-actin complex during ALD progression, were partially reverted when NXN was overexpressed in vitro [17,26,40]. This evidence places NXN as a candidate molecule to be manipulated for the treatment of diseases promoted by increased ROS levels; however, the manipulation of NXN should be imitated to the regulation of its expression, since the complete NXN deletion and mutation have also shown to be lethal and induce cardiovascular defects and abnormal bone morphology, respectively [154,155].

Although here we have summarized the multifunctionality of NXN in cellular physiology and its implication in different pathologies so far, several questions are still unresolved. For example, NXN deletion promotes the reduction of thiol proteins in neuronal cells, which strongly suggests that NXN has an important role in maintaining the homeostatic oxidation status of these proteins, as it has been shown with the status of protein phosphatase 2 catalytic subunit alpha [7]; an intriguing phenomenon encouraging to investigate whether NXN targets all thiol proteins and whether its oxidase activity is either directly on the target thiol proteins or through the participation of other oxidoreductases, such as that of the TRX family. Another obvious question is whether NXN and other TRX enzymes might be inducing a crosstalk during the regulation of some redox-regulated signaling pathways, such as TLR4/NF-κB. On the other hand, since in plant cells, NXN protects and maintains catalase enzyme in a reduced status [156], this evidence raises the question of whether NXN also reduces catalase enzyme in animal cells, and what are the associated mechanisms to decide whether NXN will play an oxidase or reductase role. 

Through immunoprecipitation analysis, it has been shown that NXN may be interacting at the same time with DVL, FZD, and PI4KII [40]; as well as, with MYD88, FLII, actin, and TLR4 [17,136], forming a binding complex of WNT/β-catenin and TLR4/MYD88/NF-κB pathways, respectively. Thus, NXN directly binds to DVL, MYD88 and FLII but indirectly forms a complex with other proteins, such as FZD, PI4KII, actin, and TLR4. This evidence arises the question of whether the proteins that directly interact with NXN, namely DVL, PFK1, PP2A, MYD88, FLII, SEC63, CAMK2A, or other proteins not yet identified, may be interacting at the same time with NXN in all tissue types or they interact and/or express in a tissue-specific manner, and what role they are playing in the formed complex. 

To date, the summarized evidence places NXN: (i) as a strong master redox regulator candidate of cell physiology, (ii) as the hub of different redox-sensitive signaling pathways, and (iii) as a key enzyme participating in the regulation of different pathologies. Therefore, the summarized data support the notion that NXN is a master regulator of cellular redox homeostasis; however, as NXN is the most recently discovered thioredoxin, only few evidences on its functioning have been revealed; for example, regarding the specific interacting domains of both NXN and its interacting proteins, as well as, its role in the regulation of several signaling pathways and associated pathologies. Thus, future research closing those gaps will definitively confirm its key role in regulating cell physiology.

## Figures and Tables

**Table 1:** Molecular interactions, signaling pathways, cellular processes, and diseases where NXN is involved in.

NXN Interaction	Signaling Pathway	Cellular Process	Disease	Experimental Model	References
**Disheveled (DVL)**	WNT/β-catenin, phosphatidylinositol 4- phosphate [PI (4) P] production	Embryogenesis and organogenesis Cell proliferation and differentiation Liver fibrogenesis	Alcoholic liver disease (ALD) Hepatocellular carcinoma (HCC) Obesity Neuroblastoma (NBL) Robinow syndrome (RS)	C57BL/6J, 129/SvJ mice, human embryonic kidney (HEK)293, murine embryonic fibroblast cell line (NIH3T3), hepatic stellate cell (HSC) and SY-SY5Y cells	[26,38,39,102,103]
**Phosphofructo kinase-1 (PFK1)**	TP53-induced glycolysis and apoptosis regulator (TIGAR)	Glycolysis Global cell metabolic state (GCMS) Cell proliferation	Retinitis pigmentosa	C57BL/6J and BALB/c mice, mouse embryonic fibroblast (MEF), HEK293	[14,44,104]
**Protein phosphatase 2A (PP2A)**	Protein kinase B (PKB) or Akt	Cell cycle progression Apoptosis Cell growth	Lung cancer	Lung cancer cell lines (NCI-H838, NCI-H1299 and NCI-H460)	[10,12,64,105]
**Myeloid differentiation primary response gene-88/Flightless-1 (MYD88/FLII)**	Nuclear factor kappa beta (NF-κB)	Inflammation Immunity Cell growth	ALD	MEFs and COS7 cells, C57BL/6J mice HEK293,NIH3T3	[9,80,106]
**FLII/ACTIN**	Nuclear receptors (NR)	Cytoskeletal dynamics Motility Contraction Adhesion Wound healing (WH) Chromatin homeostasis	ALD	C57BL/6J mice, HSC, VL17A cells	[17]
**Translocation protein SEC63 homolog (SEC63)**	IRE1α/BIP	Transport of proteins into ER Biliary cell growth Proliferation	Gastric and colorectal cancers Polycystic liver disease (PLD)	Mouse, C57BL/6J and DBA/2J	[13,107,108]
**Calcium/calmodulin-dependent protein kinase II type alpha (CAMK2A)**	None	Neuronal plasticity Development Ca2+-dependent processes	AD Autism Schizophrenia Addiction	European Conditional Mouse Mutagenesis Program (EUCOMM), Yeast-2-hybrid	[18]

## References

[1] Léveillard T., Aït-Ali N. (2017). Cell Signaling with Extracellular Thioredoxin and Thioredoxin-Like Proteins: Insight into Their Mechanisms of Action. Oxidative Med. Cell. Longev..

[2] Meyer Y., Siala W., Bashandy T., Riondet C., Vignols F., Reichheld J.P. (2008). Glutaredoxins and thioredoxins in plants. Biochim. Biophys. Acta.

[3] Funato Y., Miki H. (2007). Nucleoredoxin, a Novel Thioredoxin Family Member Involved in Cell Growth and Differentiation. Antioxid. Redox Signal..

[4] Kurooka H., Kato K., Minoguchi S., Takahashi Y., Ikeda J., Habu S., Osawa N., Buchberg A.M., Moriwaki K., Shisa H. (1997). Cloning and Characterization of the Nucleoredoxin Gene That Encodes a Novel Nuclear Protein Related to Thioredoxin. Genomics.

[5] Funato Y., Miki H. (2010). Redox regulation of Wnt signalling via nucleoredoxin. Free Radic. Res..

[6] Marchal C., Delorme-Hinoux V., Bariat L., Siala W., Belin C., Saez-Vasquez J., Riondet C., Reichheld J.-P. (2014). NTR/NRX Define a New Thioredoxin System in the Nucleus of Arabidopsis thaliana Cells. Mol. Plant.

[7] Urbainsky C., Nölker R., Imber M., Lübken A., Mostertz J., Hochgräfe F., Godoy J.R., Hanschmann E.-M., Lillig C.H. (2018). Nucleoredoxin-Dependent Targets and Processes in Neuronal Cells. Oxidative Med. Cell. Longev..

[8] Funato Y., Michiue T., Asashima M., Miki H. (2006). The Thioredoxin-Related Redox-Regulating Protein Nucleoredoxin Inhibits Wnt-Beta-Catenin Signalling through Dishevelled. Nat. Cell Biol..

[9] Hayashi T., Funato Y., Terabayashi T., Morinaka A., Sakamoto R., Ichise H., Fukuda H., Yoshida N., Miki H. (2010). Nucleoredoxin Negatively Regulates Toll-like Receptor 4 Signaling via Recruitment of Flightless-I to Myeloid Differentiation Primary Response Gene (88). J. Biol. Chem..

[10] Lechward K., Sugajska E., De Baere I., Goris J., Hemmings B.A., Zolnierowicz S. (2006). Interaction of nucleoredoxin with protein phosphatase 2A. FEBS Lett..

[11] Resjö S., Göransson O., Härndahl L., Zolnierowicz S., Manganiello V., Degerman E. (2002). Protein phosphatase 2A is the main phosphatase involved in the regulation of protein kinase B in rat adipocytes. Cell. Signal..

[12] Szymonowicz K., Oeck S., Malewicz N.M., Jendrossek V. (2018). New Insights into Protein Kinase B/Akt Signaling: Role of Localized Akt Activation and Compartment-Specific Target Proteins for the Cellular Radiation Response. Cancers.

[13] Müller L., Funato Y., Miki H., Zimmermann R. (2011). An interaction between human Sec63 and nucleoredoxin may provide the missing link between the SEC63 gene and polycystic liver disease. FEBS Lett..

[14] Funato Y., Hayashi T., Irino Y., Takenawa T., Miki H. (2013). Nucleoredoxin regulates glucose metabolism via phosphofructokinase 1. Biochem. Biophys. Res. Commun..

[15] Bartrons R., Simon-Molas H., Rodríguez-García A., Castaño E., Navarro-Sabaté À., Manzano A., Martinez-Outschoorn U.E. (2018). Fructose 2,6-Bisphosphate in Cancer Cell Metabolism. Front. Oncol..

[16] Vora S., Halper J.P., Knowles D.M. (1985). Alterations in the activity and isozymic profile of human phosphofructokinase during malignant transformation in vivo and in vitro: Transformation- and progression-linked discriminants of malignancy. Cancer Res..

[17] Alarcon-Sanchez R.B., Guerrero-Escalera D., Rosas-Madrigal S., Aparicio-Bautista D.I., Reyes-Gordillo K., Lakshman M.R., Ortiz-Fernandez A., Quezada H., Medina-Contreras O., Villa-Trevino S. (2020). Nucleoredoxin Interaction with Flightless-I/Actin Complex Is Differentially Altered in Alcoholic Liver Disease. Basic Clin. Pharmacol. Toxicol..

[18] Tran B.N., Valek L., Wilken-Schmitz A., Fuhrmann D.C., Namgaladze D., Wittig I., Tegeder I. (2021). Reduced exploratory behavior in neuronal nucleoredoxin knockout mice. Redox Biol..

[19] Hanschmann M.E., Godoy J.R., Berndt C., Hudemann C., Lillig C.H. (2013). Thioredoxins, Glutaredoxins, and Peroxiredoxins—Molecular Mechanisms and Health Significance: From Cofactors to Antioxidants to Redox Signaling. Antioxid. Redox Signal.

[20] Kallis G.B., Holmgren A. (1980). Differential reactivity of the functional sulfhydryl groups of cysteine-32 and cysteine-35 present in the reduced form of thioredoxin from Escherichia coli. J. Biol. Chem..

[21] Dyson H.J., Jeng M.-F., Tennant L.L., Slaby I., Lindell M., Cui D.-S., Kuprin A.S., Holmgren A. (1997). Effects of Buried Charged Groups on Cysteine Thiol Ionization and Reactivity in Escherichia coli Thioredoxin: Structural and Functional Characterization of Mutants of Asp 26 and Lys 57. Biochemistry.

[22] Jones D.P. (2006). Redefining Oxidative Stress. Antioxid. Redox Signal..

[23] Sies H. (2015). Oxidative stress: A concept in redox biology and medicine. Redox Biol..

[24] Mittler R. (2002). Oxidative stress, antioxidants and stress tolerance. Trends Plant Sci..

[25] Schieber M., Chandel N.S. (2014). ROS Function in Redox Signaling and Oxidative Stress. Curr. Biol..

[26] Arellanes-Robledo J., Reyes-Gordillo K., Shah R., Dominguez-Rosales J.A., Hernandez-Nazara Z.H., Ramirez F., Rojkind M., Lakshman M.R. (2013). Fibrogenic Actions of Acetaldehyde Are Beta-Catenin Dependent but Wingless Independent: A Critical Role of Nucleoredoxin and Reactive Oxygen Species in Human Hepatic Stellate Cells. Free Radic. Biol. Med..

[27] Van Amerongen R., Nusse R. (2009). Towards an Integrated View of Wnt Signaling in Development. Development.

[28] Katoh M., Katoh M. (2007). Wnt Signaling Pathway and Stem Cell Signaling Network. Clin. Cancer Res..

[29] Bhanot P., Brink M., Samos C.H., Hsieh J.-C., Wang Y., Macke J.P., Andrew D., Nathans J., Nusse R. (1996). A new member of the frizzled family from Drosophila functions as a Wingless receptor. Nature.

[30] Pinson K.I., Brennan J., Monkley S., Avery B.J., Skarnes W.C. (2000). An LDL-receptor-related protein mediates Wnt signalling in mice. Nature.

[31] Semenov M.V., Habas R., Macdonald B.T., He X. (2007). SnapShot: Noncanonical Wnt Signaling Pathways. Cell.

[32] Gao C., Chen Y.-G. (2010). Dishevelled: The hub of Wnt signaling. Cell. Signal..

[33] Boutros M., Paricio N., Strutt D., Mlodzik M. (1998). Dishevelled Activates JNK and Discriminates between JNK Pathways in Planar Polarity and wingless Signaling. Cell.

[34] MacDonald T.B., Tamai K., He X. (2009). Wnt/Beta-Catenin Signaling: Components, Mechanisms, and Diseases. Dev. Cell.

[35] Bilić J., Huang Y.-L., Davidson G., Zimmermann T., Cruciat C.-M., Bienz M., Niehrs C. (2007). Wnt Induces LRP6 Signalosomes and Promotes Dishevelled-Dependent LRP6 Phosphorylation. Science.

[36] Rharass T., Lantow M., Gbankoto A., Weiss D.G., Panakova D., Lucas S. (2017). Ascorbic Acid Alters Cell Fate Commitment of Human Neural Progenitors in a Wnt/Beta-Catenin/Ros Signaling Dependent Manner. J. Biomed. Sci..

[37] Kajla S., Mondol A.S., Nagasawa A., Zhang Y., Kato M., Matsuno K., Yabe-Nishimura C., Kamata T. (2012). A Crucial Role for Nox 1 in Redox-Dependent Regulation of Wnt-Beta-Catenin Signaling. FASEB J..

[38] Funato Y., Michiue T., Terabayashi T., Yukita A., Danno H., Asashima M., Miki H. (2008). Nucleoredoxin regulates the Wnt/planar cell polarity pathway inXenopus. Genes Cells.

[39] Bahn J.Y., Lee K.P., Lee S.M., Choi J.Y., Seo Y.S., Kwon K.S. (2015). Nucleoredoxin Promotes Adipogenic Differentiation through Regulation of Wnt/Beta-Catenin Signaling. J. Lipid Res..

[40] Arellanes-Robledo J., Reyes-Gordillo K., Ibrahim J., Leckey L., Shah R., Lakshman M.R. (2018). Ethanol targets nucleoredoxin/dishevelled interactions and stimulates phosphatidylinositol 4-phosphate production in vivo and in vitro. Biochem. Pharmacol..

[41] Kanai S., Shimada T., Narita T., Okabayashi K. (2019). Phosphofructokinase-1 Subunit Composition and Activity in the Skeletal Muscle, Liver, and Brain of Dogs. J. Vet. Med. Sci..

[42] Zancan P., Almeida F.V., Faber-Barata J., Dellias J.M., Sola-Penna M. (2007). Fructose-2,6-bisphosphate counteracts guanidinium chloride-, thermal-, and ATP-induced dissociation of skeletal muscle key glycolytic enzyme 6-phosphofructo-1-kinase: A structural mechanism for PFK allosteric regulation. Arch. Biochem. Biophys..

[43] Bartrons R., Rodríguez-García A., Simon-Molas H., Castaño E., Manzano A., Navarro-Sabaté À. (2018). The potential utility of PFKFB3 as a therapeutic target. Expert Opin. Ther. Targets.

[44] Yalcin A., Telang S., Clem B., Chesney J. (2009). Regulation of Glucose Metabolism by 6-Phosphofructo-2-Kinase/Fructose-2,6-Bisphosphatases in Cancer. Exp. Mol. Pathol..

[45] Hennipman A., Smits J., Van Oirschot B., Van Houwelingen J., Rijksen G., Neyt J., Van Unnik J., Staal G. (1987). Glycolytic Enzymes in Breast Cancer, Benign Breast Disease and Normal Breast Tissue. Tumor Biol..

[46] Moreno-Sánchez R., Rodriguez-Enriquez S., Marín-Hernández Á., Saavedra E. (2007). Energy metabolism in tumor cells. FEBS J..

[47] Bensaad K., Tsuruta A., Selak M.A., Vidal M.N.C., Nakano K., Bartrons R., Gottlieb E., Vousden K.H. (2006). TIGAR, a p53-Inducible Regulator of Glycolysis and Apoptosis. Cell.

[48] Bensaad K., Cheung E.C., Vousden K.H. (2009). Modulation of intracellular ROS levels by TIGAR controls autophagy. EMBO J..

[49] Lui V.W.Y., Lau C.P.Y., Cheung C.S.F., Ho K., Ng M.H.L., Cheng S.H., Hong B., Tsao S.-W., Tsang C.M., Lei K.I.K. (2010). An RNA-directed nucleoside anti-metabolite, 1-(3-C-ethynyl-beta-d-ribo-pentofuranosyl)cytosine (ECyd), elicits antitumor effect via TP53-induced Glycolysis and Apoptosis Regulator (TIGAR) downregulation. Biochem. Pharmacol..

[50] Yi W., Clark P.M., Mason D.E., Keenan M.C., Hill C., Goddard W.A., Peters E.C., Driggers E.M., Hsieh-Wilson L.C. (2012). Phosphofructokinase 1 Glycosylation Regulates Cell Growth and Metabolism. Science.

[51] Eichhorn P.J., Creyghton M.P., Bernards R. (2009). Protein phosphatase 2A regulatory subunits and cancer. Biochim. Biophys. Acta.

[52] Groves R.M., Hanlon N., Turowski P., Hemmings B.A., Barford D. (1999). The Structure of the Protein Phosphatase 2a Pr65/a Subunit Reveals the Conformation of Its 15 Tandemly Repeated Heat Motifs. Cell.

[53] Guo F., Stanevich V., Wlodarchak N., Sengupta R., Jiang L., Satyshur K.A., Xing Y. (2014). Structural Basis of Pp2a Activation by Ptpa, an Atp-Dependent Activation Chaperone. Cell Res..

[54] Wlodarchak N., Xing Y. (2016). PP2A as a master regulator of the cell cycle. Crit. Rev. Biochem. Mol. Biol..

[55] Ugi S., Imamura T., Ricketts W., Olefsky J.M. (2002). Protein Phosphatase 2A Forms a Molecular Complex with Shc and Regulates Shc Tyrosine Phosphorylation and Downstream Mitogenic Signaling. Mol. Cell. Biol..

[56] McCubrey A.J., Steelman L.S., Chappell W.H., Abrams S.L., Wong E.W., Chang F., Lehmann B., Terrian D.M., Milella M., Tafuri A. (2007). Roles of the Raf/Mek/Erk Pathway in Cell Growth, Malignant Transformation and Drug Resistance. Biochim. Biophys. Acta.

[57] Meng G., Wang W., Chai K., Yang S., Li F., Jiang K. (2015). Combination Treatment with Triptolide and Hydroxycamptothecin Synergistically Enhances Apoptosis in A549 Lung Adenocarcinoma Cells through Pp2a-Regulated Erk, P38 Mapks and Akt Signaling Pathways. Int. J. Oncol..

[58] Shi Y. (2009). Serine/Threonine Phosphatases: Mechanism through Structure. Cell.

[59] Arriazu E., Pippa R., Odero M.D. (2016). Protein Phosphatase 2a as a Therapeutic Target in Acute Myeloid Leukemia. Front. Oncol..

[60] Humphrey S.J., James D.E. (2012). Uncaging Akt. Sci. Signal.

[61] Yang J., Wu Z., Renier N., Simon D.J., Uryu K., Park D., Greer P.A., Tournier C., Davis R.J., Tessier-Lavigne M. (2015). Pathological Axonal Death through a MAPK Cascade that Triggers a Local Energy Deficit. Cell.

[62] Wang W.Y., Lin K.T., Chen S.C., Gu D.L., Chen C.F., Tu P.H., Jou Y.S. (2013). Overexpressed-Eif3i Interacted and Activated Oncogenic Akt1 Is a Theranostic Target in Human Hepatocellular Carcinoma. Hepatology.

[63] Dennis M.D., Coleman C.S., Berg A., Jefferson L.S., Kimball S.R. (2014). REDD1 enhances protein phosphatase 2A–mediated dephosphorylation of Akt to repress mTORC1 signaling. Sci. Signal..

[64] Lei N., Peng B., Zhang J.-Y. (2014). CIP2A regulates cell proliferation via the AKT signaling pathway in human lung cancer. Oncol. Rep..

[65] Ohama T., Brautigan D.L. (2010). Endotoxin Conditioning Induces VCP/p97-mediated and Inducible Nitric-oxide Synthase-dependent Tyr284 Nitration in Protein Phosphatase 2A. J. Biol. Chem..

[66] Dagnell M., Pace P.E., Cheng Q., Frijhoff J., Östman A., Arnér E., Hampton M.B., Winterbourn C.C. (2017). Thioredoxin reductase 1 and NADPH directly protect protein tyrosine phosphatase 1B from inactivation during H2O2 exposure. J. Biol. Chem..

[67] Lord K., Hoffman-Liebermann B., Liebermann D. (1990). Nucleotide sequence and expression of a cDNA encoding MyD88, a novel myeloid differentiation primary response gene induced by IL6. Oncogene.

[68] Kawai T., Adachi O., Ogawa T., Takeda K., Akira S. (1999). Unresponsiveness of MyD88-Deficient Mice to Endotoxin. Immunity.

[69] Deguine J., Barton G.M. (2014). MyD88: A central player in innate immune signaling. F1000Prime Rep..

[70] O’Neill L.A.J., Golenbock D., Bowie A.G. (2013). The history of Toll-like receptors—redefining innate immunity. Nat. Rev. Immunol..

[71] Liang Y., Zhou Y., Shen P. (2004). Nf-Kappab and Its Regulation on the Immune System. Cell Mol. Immunol..

[72] Pereira G.S., Oakley F. (2008). Nuclear Factor-Kappab1: Regulation and Function. Int. J. Biochem. Cell Biol..

[73] Kawai T., Akira S. (2011). Toll-like Receptors and Their Crosstalk with Other Innate Receptors in Infection and Immunity. Immunity.

[74] Anwar M.A., Basith S., Choi S. (2013). Negative regulatory approaches to the attenuation of Toll-like receptor signaling. Exp. Mol. Med..

[75] O’Neill L.A., Fitzgerald K., Bowie A. (2003). The Toll–IL-1 receptor adaptor family grows to five members. Trends Immunol..

[76] Bieghs V., Trautwein C. (2014). Innate immune signaling and gut-liver interactions in non-alcoholic fatty liver disease. Hepatobiliary Surg. Nutr..

[77] Yin H., Huang L., Ouyang T., Chen L. (2018). Baicalein Improves Liver Inflammation in Diabetic Db/Db Mice by Regulating Hmgb1/Tlr4/Nf-Kappab Signaling Pathway. Int. Immunopharmacol..

[78] Liew F.Y., Xu D., Brint E., O’Neill L. (2005). Negative regulation of Toll-like receptor-mediated immune responses. Nat. Rev. Immunol..

[79] Wang T., Gu S., Ronni T., Du Y.-C., Chen X. (2005). In Vivo Dual-Tagging Proteomic Approach in Studying Signaling Pathways in Immune Response. J. Proteome Res..

[80] Ruzehaji N., Mills S.J., Melville E., Arkell R., Fitridge R., Cowin A.J. (2013). The Influence of Flightless I on Toll-Like-Receptor-Mediated Inflammation in a Murine Model of Diabetic Wound Healing. BioMed Res. Int..

[81] Wang T., Chuang T.-H., Ronni T., Gu S., Du Y.-C., Cai H., Tsung-Hsien C., Yin H.L., Chen X. (2006). Flightless I Homolog Negatively Modulates the TLR Pathway. J. Immunol..

[82] Davy D., Campbell H.D., Fountain S., De Jong D., Crouch M.F. (2001). The flightless I protein colocalizes with actin- and microtubule-based structures in motile Swiss 3T3 fibroblasts: Evidence for the involvement of PI 3-kinase and Ras-related small GTPases. J. Cell Sci..

[83] Lee Y.-H., Campbell H.D., Stallcup M.R. (2004). Developmentally Essential Protein Flightless I Is a Nuclear Receptor Coactivator with Actin Binding Activity. Mol. Cell. Biol..

[84] Archer S., Claudianos C., Campbell H.D. (2005). Evolution of the gelsolin family of actin-binding proteins as novel transcriptional coactivators. BioEssays.

[85] Jeong K.W. (2014). Flightless I (Drosophila) Homolog Facilitates Chromatin Accessibility of the Estrogen Receptor Alpha Target Genes in Mcf-7 Breast Cancer Cells. Biochem. Biophys. Res. Commun..

[86] Li X., Sun S., Appathurai S., Sundaram A., Plumb R., Mariappan M. (2020). A Molecular Mechanism for Turning Off IRE1α Signaling during Endoplasmic Reticulum Stress. Cell Rep..

[87] Walter P., Ron D. (2011). The Unfolded Protein Response: From Stress Pathway to Homeostatic Regulation. Science.

[88] Davila S., Furu L., Gharavi A.G., Tian X., Onoe T., Qian Q., Li A., Cai Y., Kamath P.S., King B.F. (2004). Mutations in SEC63 cause autosomal dominant polycystic liver disease. Nat. Genet..

[89] Lang S., Benedix J., Fedeles S.V., Schorr S., Schirra C., Schäuble N., Jalal C., Greiner M., Haßdenteufel S., Tatzelt J. (2012). Differential effects of Sec61α-, Sec62- and Sec63-depletion on transport of polypeptides into the endoplasmic reticulum of mammalian cells. J. Cell Sci..

[90] Rapoport T.A. (2007). Protein translocation across the eukaryotic endoplasmic reticulum and bacterial plasma membranes. Nature.

[91] Jermy A., Willer M., Davis E., Wilkinson B.M., Stirling C.J. (2006). The Brl Domain in Sec63p Is Required for Assembly of Functional Endoplasmic Reticulum Translocons. J. Biol. Chem..

[92] Conti J.B., Devaraneni P.K., Yang Z., David L.L., Skach W.R. (2015). Cotranslational Stabilization of Sec62/63 within the Er Sec61 Translocon Is Controlled by Distinct Substrate-Driven Translocation Events. Mol. Cell.

[93] Liu B.X., Murray K.D. (2012). Neuronal Excitability and Calcium/Calmodulin-Dependent Protein Kinase Type Ii: Location, Location, Location. Epilepsia.

[94] Hell J.W. (2014). CaMKII: Claiming Center Stage in Postsynaptic Function and Organization. Neuron.

[95] Lisman J., Yasuda R., Raghavachari S. (2012). Mechanisms of CaMKII action in long-term potentiation. Nat. Rev. Neurosci..

[96] Hanson I.P., Schulman H. (1992). Neuronal Ca2+/Calmodulin-Dependent Protein Kinases. Annu. Rev. Biochem..

[97] Erondu E.N., Kennedy M.B. (1985). Regional Distribution of Type Ii Ca2+/Calmodulin-Dependent Protein Kinase in Rat Brain. J. Neurosci..

[98] Soderling R.T., Chang B., Brickey D. (2001). Cellular Signaling through Multifunctional Ca^2+^/Calmodulin-Dependent Protein Kinase Ii. J. Biol. Chem..

[99] Coultrap S.J., Freund R.K., O’Leary H., Sanderson J.L., Roche K., Dell’Acqua M.L., Bayer K.U. (2014). Autonomous CaMKII Mediates Both LTP and LTD Using a Mechanism for Differential Substrate Site Selection. Cell Rep..

[100] Giese P.K., Fedorov N.B., Filipkowski R.K., Silva A.J. (1998). Autophosphorylation at Thr286 of the Alpha Calcium-Calmodulin Kinase Ii in Ltp and Learning. Science.

[101] Jaillard C., Ouechtati F., Clérin E., Millet-Puel G., Corsi M., Aït-Ali N., Blond F., Chevy Q., Gales L., Farinelli M. (2021). The metabolic signaling of the nucleoredoxin-like 2 gene supports brain function. Redox Biol..

[102] Valek L., Tegeder I. (2021). Nucleoredoxin Knockdown in SH-SY5Y Cells Promotes Cell Renewal. Antioxidants.

[103] White J.J., Mazzeu J., Coban-Akdemir Z., Bayram Y., Bahrambeigi V., Hoischen A., van Bon B.W., Gezdirici A., Gulec E.Y., Ramond F. (2017). WNT Signaling Perturbations Underlie the Genetic Heterogeneity of Robinow Syndrome. Am. J. Hum. Genet..

[104] Clérin E., Marussig M., Sahel J.-A., Léveillard T. (2020). Metabolic and Redox Signaling of the Nucleoredoxin-Like-1 Gene for the Treatment of Genetic Retinal Diseases. Int. J. Mol. Sci..

[105] Janssens V., Goris J. (2001). Protein Phosphatase 2a: A Highly Regulated Family of Serine/Threonine Phosphatases Implicated in Cell Growth and Signalling. Biochem. J..

[106] Hirota K., Matsui M., Murata M., Takashima Y., Cheng F.S., Itoh T., Fukuda K., Yodoi J. (2000). Nucleoredoxin, Glutaredoxin, and Thioredoxin Differentially Regulate Nf-Kappab, Ap-1, and Creb Activation in Hek293 Cells. Biochem. Biophys. Res. Commun..

[107] Mori Y., Sato F., Selaru F.M., Olaru A., Perry K., Kimos M.C., Tamura G., Matsubara N., Wang S., Xu Y. (2002). Instabilotyping reveals unique mutational spectra in microsatellite-unstable gastric cancers. Cancer Res..

[108] Casper M., Weber S.N., Kloor M., Müllenbach R., Grobholz R., Lammert F., Zimmer V. (2012). Hepatocellular carcinoma as extracolonic manifestation of Lynch syndrome indicatesSEC63as potential target gene in hepatocarcinogenesis. Scand. J. Gastroenterol..

[109] Asmat U., Abad K., Ismail K. (2015). Diabetes mellitus and oxidative stress—A concise review. Saudi Pharm. J..

[110] Association, American Diabetes (2014). Diagnosis and Classification of Diabetes Mellitus. Diabetes Care.

[111] Babaya N., Ikegami H., Fujisawa T., Nojima K., Itoi-Babaya M., Inoue K., Ohno T., Shibata M., Ogihara T. (2005). Susceptibility to streptozotocin-induced diabetes is mapped to mouse chromosome 11. Biochem. Biophys. Res. Commun..

[112] Suryavanshi S.V., Kulkarni Y.A. (2017). Nf-Kappabeta: A Potential Target in the Management of Vascular Complications of Diabetes. Front. Pharmacol..

[113] Chooi C.Y., Ding C., Magkos F. (2019). The Epidemiology of Obesity. Metabolism.

[114] Blüher M. (2019). Obesity: Global epidemiology and pathogenesis. Nat. Rev. Endocrinol..

[115] Spiegelman B.M., Flier J.S. (2001). Obesity and the Regulation of Energy Balance. Cell.

[116] Xu H., Barnes G.T., Yang Q., Tan G., Yang D., Chou C.J., Sole J., Nichols A., Ross J.S., Tartaglia L.A. (2003). Chronic Inflammation in Fat Plays a Crucial Role in the Development of Obesity-Related Insulin Resistance. J. Clin. Investig..

[117] Rosen D.E., Spiegelman B.M. (2000). Molecular Regulation of Adipogenesis. Annu. Rev. Cell Dev. Biol..

[118] Moldes M., Zuo Y., Morrison R.F., Silva D., Park B.-H., Liu J., Farmer S.R. (2003). Peroxisome-proliferator-activated receptor γ suppresses Wnt/β-catenin signalling during adipogenesis. Biochem. J..

[119] Ross S.E., Hemati N., Longo K.A., Bennett C.N., Lucas P.C., Erickson R.L., MacDougald O.A. (2000). Inhibition of Adipogenesis by Wnt Signaling. Science.

[120] Jocken J.W.E., Langin D., Smit E., Saris W.H.M., Valle C., Hul G.B., Holm C., Arner P., Blaak E.E. (2007). Adipose Triglyceride Lipase and Hormone-Sensitive Lipase Protein Expression Is Decreased in the Obese Insulin-Resistant State. J. Clin. Endocrinol. Metab..

[121] Aron-Wisnewsky J., Tordjman J., Poitou C., Darakhshan F., Hugol D., Basdevant A., Aissat A., Guerre-Millo M., Clément K. (2009). Human Adipose Tissue Macrophages: M1 and M2 Cell Surface Markers in Subcutaneous and Omental Depots and after Weight Loss. J. Clin. Endocrinol. Metab..

[122] Mathys H., Davila-Velderrain J., Peng Z., Gao F., Mohammadi S., Young J.Z., Menon M., He L., Abdurrob F., Jiang X. (2019). Single-cell transcriptomic analysis of Alzheimer’s disease. Nature.

[123] Duthey B. (2013). Background Paper 6.11: Alzheimer Disease and Other Dementias. Public Health Approach Innov..

[124] Jaillard C., Mouret A., Niepon M., Clérin E., Yang Y., Lee-Rivera I., Aït-Ali N., Millet-Puel G., Cronin T., Sedmak T. (2012). Nxnl2 Splicing Results in Dual Functions in Neuronal Cell Survival and Maintenance of Cell Integrity. Hum. Mol. Genet..

[125] Müller T., Concannon C.G., Ward M.W., Walsh C.M., Tirniceriu A.L., Tribl F., Kögel D., Prehn J.H.M., Egensperger R. (2007). Modulation of Gene Expression and Cytoskeletal Dynamics by the Amyloid Precursor Protein Intracellular Domain (Aicd). Mol. Biol. Cell.

[126] Xicoy H., Wieringa B., Martens G.J.M. (2017). The SH-SY5Y cell line in Parkinson’s disease research: A systematic review. Mol. Neurodegener..

[127] Maris M.J., Hogarty M.D., Bagatell R., Cohn S.L. (2007). Neuroblastoma. Lancet.

[128] Lamsa K., Irvine E.E., Giese K.P., Kullmann D.M. (2007). Nmda Receptor-Dependent Long-Term Potentiation in Mouse Hippocampal Interneurons Shows a Unique Dependence on Ca(2+)/Calmodulin-Dependent Kinases. J. Physiol..

[129] Mijakowska Z., Łukasiewicz K., Ziółkowska M., Lipiński M., Trąbczyńska A., Matuszek Ż., Łęski S., Radwanska K. (2017). Autophosphorylation of Alpha Isoform of Calcium/Calmodulin-Dependent Kinase Ii Regulates Alcohol Addiction-Related Behaviors. Addict. Biol..

[130] Duman R.S. (2004). Neural plasticity: Consequences of stress and actions of antidepressant treatment. Dialog. Clin. Neurosci..

[131] Miller A.M., Horiguchi N., Jeong W.-I., Radaeva S., Gao B. (2011). Molecular Mechanisms of Alcoholic Liver Disease: Innate Immunity and Cytokines. Alcohol. Clin. Exp. Res..

[132] Rehm J., Taylor B., Mohapatra S., Irving H., Baliunas D., Patra J., Roerecke M. (2010). Alcohol as a risk factor for liver cirrhosis: A systematic review and meta-analysis. Drug Alcohol Rev..

[133] Jiang Y., Zhang T., Kusumanchi P., Han S., Yang Z., Liangpunsakul S. (2020). Alcohol Metabolizing Enzymes, Microsomal Ethanol Oxidizing System, Cytochrome P450 2E1, Catalase, and Aldehyde Dehydrogenase in Alcohol-Associated Liver Disease. Biomedicines.

[134] Linhart K., Bartsch H., Seitz H.K. (2014). The role of reactive oxygen species (ROS) and cytochrome P-450 2E1 in the generation of carcinogenic etheno-DNA adducts. Redox Biol..

[135] Fujino G., Noguchi T., Takeda K., Ichijo H. (2006). Thioredoxin and protein kinases in redox signaling. Semin. Cancer Biol..

[136] Arellanes-Robledo J., Ibrahim J., Reyes-Gordillo K., Shah R., Leckey L., Lakshman M.R. (2020). Flightless-I is a potential biomarker for the early detection of alcoholic liver disease. Biochem. Pharmacol..

[137] Fedeles S.V., So J.-S., Shrikhande A., Lee S.H., Gallagher A.-R., Barkauskas C.E., Somlo S., Lee A.-H. (2015). Sec63 and Xbp1 regulate IRE1α activity and polycystic disease severity. J. Clin. Investig..

[138] Fedeles S.V., Gallagher A.-R., Somlo S. (2014). Polycystin-1: A master regulator of intersecting cystic pathways. Trends Mol. Med..

[139] Janssen M.J., Salomon J., Morsche R.H.M.T., Drenth J.P.H. (2012). Loss of Heterozygosity Is Present in SEC63 Germline Carriers with Polycystic Liver Disease. PLoS ONE.

[140] Dryja T.P., McGee T.L., Reichel E., Hahn L.B., Cowley G.S., Yandell D.W., Sandberg M.A., Berson E.L. (1990). A point mutation of the rhodopsin gene in one form of retinitis pigmentosa. Nature.

[141] Marlhens F., Bareil C., Griffoin J., Zrenner E., Amalric P., Eliaou C., Liu S., Harris E., Redmond T.M., Arnaud B. (1997). Mutations in Rpe65 Cause Leber’s Congenital Amaurosis. Nat. Genet..

[142] Redmond T.M.., Yu S., Lee E., Bok D., Hamasaki D., Chen N., Goletz P., Ma J.-X., Crouch R.K., Pfeifer K. (1998). Rpe65 is necessary for production of 11-cis-vitamin A in the retinal visual cycle. Nat. Genet..

[143] Sahel J.-A., Léveillard T., Picaud S., Dalkara D., Marazova K., Safran A., Paques M., Duebel J., Roska B., Mohand-Said S. (2013). Functional Rescue of Cone Photoreceptors in Retinitis Pigmentosa. Graefe Arch. Clin. Exp. Ophthalmol..

[144] Mei X., Chaffiol A., Kole C., Yang Y., Millet-Puel G., Clérin E., Aït-Ali N., Bennett J., Dalkara D., Sahel J. (2016). The Thioredoxin Encoded by the Rod-Derived Cone Viability Factor Gene Protects Cone Photoreceptors against Oxidative Stress. Antioxid. Redox Signal..

[145] Bowes C., Li T., Danciger M., Baxter L.C., Applebury M.L., Farber D.B. (1990). Retinal Degeneration in the Rd Mouse Is Caused by a Defect in the Β Subunit of Rod Cgmp-Phosphodiesterase. Nature.

[146] Chang G.-Q., Hao Y., Wong F. (1993). Apoptosis: Final Common Pathway of Photoreceptor Death in Rd, Rds, and Mutant Mice. Neuron.

[147] Komeima K., Rogers B.S., Lu L., Campochiaro P.A. (2006). Antioxidants reduce cone cell death in a model of retinitis pigmentosa. Proc. Natl. Acad. Sci. USA.

[148] Portera-Cailliau C., Sung C.H., Nathans J., Adler R. (1994). Apoptotic photoreceptor cell death in mouse models of retinitis pigmentosa. Proc. Natl. Acad. Sci. USA.

[149] Cronin T., Raffelsberger W., Lee-Rivera I., Jaillard C., Niepon M.-L., Kinzel B., Clérin E., Petrosian A., Picaud S., Poch O. (2010). The disruption of the rod-derived cone viability gene leads to photoreceptor dysfunction and susceptibility to oxidative stress. Cell Death Differ..

[150] Warman M.L., Cormier-Daire V., Hall C., Krakow D., Lachman R., LeMerrer M., Mortier G., Mundlos S., Nishimura G., Rimoin D.L. (2011). Nosology and classification of genetic skeletal disorders: 2010 revision. Am. J. Med. Genet. Part A.

[151] Patton M.A., Afzal A.R. (2002). Robinow Syndrome. J. Med. Genet..

[152] Van Bokhoven H., Celli J., Kayserili H., Van Beusekom E., Balci S., Brussel W., Skovby F., Kerr B., Percin E.F., Akarsu N. (2000). Mutation of the gene encoding the ROR2 tyrosine kinase causes autosomal recessive Robinow syndrome. Nat. Genet..

[153] Gu S., Yuan B., Campbell I., Beck C.R., Carvalho C.M., Nagamani S.C., Erez A., Patel A., Bacino C.A., Shaw C.A. (2015). Alu-mediated diverse and complex pathogenic copy-number variants within human chromosome 17 at p13.3. Hum. Mol. Genet..

[154] Boles K.M., Wilkinson B.M., Wilming L.G., Liu B., Probst F.J., Harrow J., Grafham D., Hentges K.E., Woodward L.P., Maxwell A. (2009). Discovery of Candidate Disease Genes in Enu-Induced Mouse Mutants by Large-Scale Sequencing, Including a Splice-Site Mutation in Nucleoredoxin. PLoS Genet..

[155] Funato Y., Terabayashi T., Sakamoto R., Okuzaki D., Ichise H., Nojima H., Yoshida N., Miki H. (2010). Nucleoredoxin Sustains Wnt/Beta-Catenin Signaling by Retaining a Pool of Inactive Dishevelled Protein. Curr. Biol..

[156] Kneeshaw S., Keyani R., Delorme-Hinoux V., Imrie L., Loake G.J., Le Bihan T., Reichheld J.-P., Spoel S.H. (2017). Nucleoredoxin guards against oxidative stress by protecting antioxidant enzymes. Proc. Natl. Acad. Sci. USA.

